# Chirality‐Induced Hydroxyapatite Manipulates Enantioselective Bone‐Implant Interactions Toward Ameliorative Osteoporotic Osseointegration

**DOI:** 10.1002/advs.202411602

**Published:** 2024-12-31

**Authors:** Liang Yang, Jinzhou Du, Shengyang Jin, Shuyi Yang, Zhaowei Chen, Shiyang Yu, Cunyi Fan, Chao Zhou, Hongjiang Ruan

**Affiliations:** ^1^ Department of Orthopedics Shanghai Sixth People's Hospital Affiliated to Shanghai Jiao Tong University School of Medicine Shanghai 200233 P. R. China; ^2^ Shanghai Engineering Research Center for Orthopedic Material Innovation and Tissue Regeneration Shanghai 200233 P. R. China; ^3^ Department of Orthopaedics The First Affiliated Hospital of Soochow University Suzhou 215006 P. R. China; ^4^ Department of Radiology Zhongshan Hospital Fudan University Shanghai 200032 P. R. China

**Keywords:** chirality, enantioselectivity, hydroxyapatite, immunomodulation, osteoporotic osseointegration

## Abstract

Inspired by the fundamental attribute of chirality in nature, chiral‐engineered biomaterials now represent a groundbreaking frontier in biomedical fields. However, the integration of chirality within inorganic materials remains a critical challenge and developments of chirality‐induced bionic bone implants are still in infancy. In this view, novel chiral hydroxyapatite (CHA) coated Ti alloys are successfully synthesized by a sophisticated chiral molecule‐induced self‐assembly method for the first time. The obtained samples are characterized by stereospecific L‐/D‐/Rac‐chiral hierarchical morphology, nanotopography rough surfaces, improved hydrophilicity, and bioactivity. Following implantation into rat femoral condyle defects, the distinct stereospecific chiral hierarchical structures exhibit highly enantioselective bone‐implants interactions, wherein the left‐handed chirality of L‐CHA strongly promotes osteoporotic osseointegration and vice versa for right‐handed chirality of D‐CHA. Consistently, in vitro assays further validate the superior enantiomer‐dependent osteoporotic osseointegration ability of L‐CHA, mainly by manipulating desired immunomodulation coupled with enhanced neurogenesis, angiogenesis, and osteogenesis. Moreover, as analyzed by transcriptomic RNA‐seq, a new discovery of down‐regulated IL‐17 signaling pathway is considered predominately responsible for the desired immunomodulation ability of L‐CHA. These results provide new insights into biological multifunctionality and mechanism underlying L‐chirality's roles for bone healing, thus may inspiring developments of new generation of chiral biomaterials.

## Graphical abstract

This study highlights the superior chirality‐dependent osteoporotic osseointegration ability of left‐handed chiral hydroxyapatite (L‐CHA) by down‐regulating IL‐17 signaling pathway to manipulate desired immunoregulation coupled with enhanced neurovascularization, which provides new insights for better understanding L‐chirality's roles on dictating bone regeneration and hence may inspire the developments of new generation of chiral engineered biomaterials.

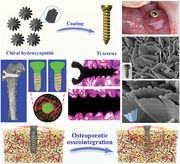

## 1 Introduction

With the population aging, the elevated morbidity of bone fracture boosts the demand for orthopedic implantation.^[^ ^1^ ^]^ Moreover, due to limited self‐healing capacity of degenerative bone, traditional bone grafting therapies become less viable and, therefore, innovative solutions have been extensively explored for treating refractory bone defects under pathological conditions such as osteoporosis.^[^ ^2^, ^3^, ^4^, ^5^ ^]^ Correspondingly, the developments of bionic grafts by simulating structure and composition of natural bone represent a highly promising strategy in bone tissue engineering.^[^ ^6^, ^7^ ^]^ Currently, various sophisticated biomaterials possessing biomimetic physico‐chemical properties have been designed to match natural bone, while one critical challenge remaining is the integration of chirality within biomaterials since chirality is exactly a fundamental attribute of nature and life science from micro‐to‐macroscopic levels.^[^ ^8^ ^]^

Actually, chirality is ubiquitous in all species and plays essential roles in living matter including DNA, carbohydrates, amino acids, proteins, organelles, and complex organs.^[^ ^9^ ^]^ More importantly, the homochiral selectivity during evolution comprehensively modulates multiple biological processes including cellular morphological changes, immune response, polarization reactions, self‐assembly, enantioselective catalysis, tissue repair et al.^[^ ^10^ ^]^ Consequently, the intrinsic chirality pertains to organic collagen fibers and then exquisitely mediates the helical self‐assembly mineralization of natural bone, indicating minerals in bone tissues are thoroughly characterized by chiral hierarchical structures at all levels.^[^ ^11^ ^]^ On this basis, the inherent chiral configurations in biomolecules generate unlimited potentialities to interact with artificial chiral materials. Therefore, the role of chirality in biomimetic implants has gained much attention and the development of chiral‐engineered biomaterials is becoming a groundbreaking frontier in biomedical fields especially for bone repair.^[^ ^9^, ^12^, ^13^ ^]^

Among commercially available bone biomaterials, hydroxyapatite (HA), as the major inorganic constituent of natural bone, is frequently used to promote bone regeneration/osseointegration for its well‐established osteoconductivity and biocompatibility.^[^ ^14^ ^]^ Nevertheless, the clinical efficacy of synthetic HA reveals somewhat unsatisfactory especially for repairing bone under pathological environments such as osteoporosis, diabetes, and infection. This phenomenon may be attributed to the fact that commercially available HA is mostly synthesized based on simple Ca/P stoichiometry and self‐assembly mineralization methodology,^[^ ^15^, ^16^, ^17^ ^]^ but neglects the stereospecific chiral hierarchical structure of natural HA, thus failing to exhaustively simulate the intrinsic topological structure and regenerative properties of natural bone. Up to now, researches concerning chirality‐inspired biomaterials and their regenerative potential in tissue engineering remain in infancy, particularly investigations about chiral inorganic materials and their interactions with bone are few performed.^[^ ^9^, ^12^, ^18^ ^]^

To address this issue, we have been devoted to explore chirality‐induced bionic implants by using chiral arginine‐modified electrospun membrane or hierarchical chiral calcium silicate hydrate film recently, and the results demonstrate L‐chiral (Left‐handed) bioscaffolds are able to strongly promote tendon or tendon‐to‐bone healing.^[^ ^19^, ^20^ ^]^ Furthermore, we thoroughly analyzed nine levels of hierarchical chiral structure of natural bone from atomic to macroscopic scales for the first time^[^ ^11^ ^]^ and then successfully synthesized chiral inorganic mesostructured HA, wherein L‐chiral HA exhibited desired enantioselective interactions with MSCs/osteoblasts to promote osteogenesis.^[^ ^21^, ^22^ ^]^ Likewise, scholars also discovered ECM‐mimetic L‐chiral hydrogels induced highly enantioselective actions on MSCs to accelerate cell adhesion, proliferation, osteogenic differentiation, and biomineralization, providing solid foundations to develop chirality‐induced biomaterials for bone tissue engineering.^[^ ^23^, ^24^ ^]^ Nevertheless, the exact bioeffects and underlying mechanism of chirality in regulating bone healing remain largely unclear, because bone regeneration is indeed composed of chronological and dynamical events coordinated by various cells from multiple systems.^[^ ^25^ ^]^

**Figure 1:**
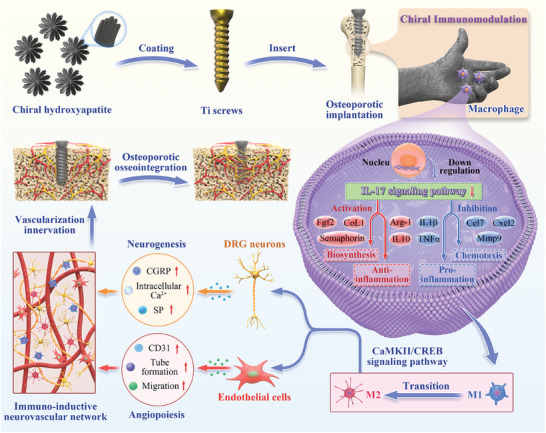
Schematic illustration of L‐CHA coated Ti which manipulates desired immunomodulation coupled with enhanced neurogenesis and angiogenesis toward ameliorative osteoporotic osseointegration.

Hence, based on our past studies related to chirality‐induced bionic materials,^[^ ^19^, ^20^, ^21^, ^22^ ^]^ for the first time, present study intended to explore the superior therapeutic potentials and underlying mechanism of chiral HA (CHA) for bone healing. Thus, a sophisticated chiral molecule‐induced self‐assembly method was applied to synthesize chiral (L‐, D‐, Racemic‐) and achiral HA, which were then deposited on Ti alloys to generate stereospecific enantioselective interface for biological investigations. Rat femoral condyle defects under osteoporotic conditions were performed to clarify the enantiomer‐dependent osseointegration induced by CHA. The bioeffects of CHA on modulating bone regeneration events were comprehensively examined to determine the optimal chiral structure of HA, and RNA‐seq was further carried out to analyze the underlying mechanism, which may provide new insights into chirality‐induced functional bone healing and inspire the developments of new generation of chiral engineered biomaterials (**Figure** **1**).

## 2 Results and Discussion

### 2.1 Characterization of CHA Coated Ti

**Figure 2:**
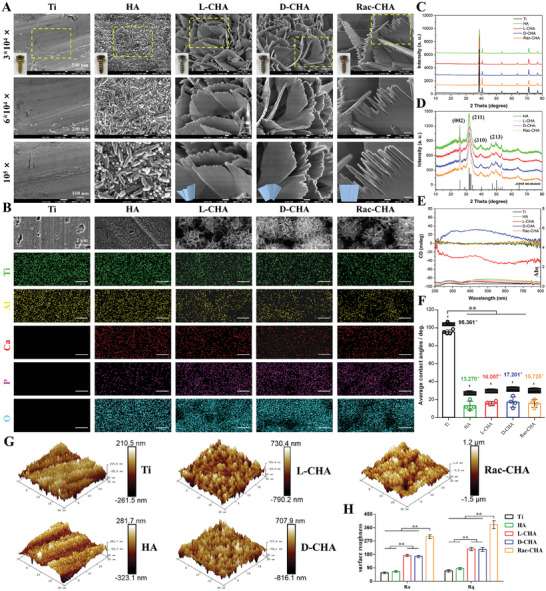
Physicochemical properties characterizations of CHA coated Ti. A) Macroscopical view and representative SEM images of samples at various magnifications. The areas marked by yellow box are magnified for observation. B) SEM‐EDS mapping of samples including Ti, Al, Ca, P, and O elements. C,D) XRD patterns of (C) CHA‐coated Ti and (D) synthesized powder of CHA coating. E) Chirality of samples determined by UV–vis and CD spectra. F) Surface hydrophilicity of samples quantified by contact angles. G,H) Surface roughness of samples examined by AFM and analyzed based on Ra and Rq parameters. *n* = 4. ^**^ *p* < 0.01.

As shown in **Figure** **2**A, the macroscopical color of Ti screws transformed from yellow (pristine Ti) to brown (HA) and grey‐white (L‐CHA, D‐CHA, Rac‐CHA) due to deposition of CHA coating following hydrothermal reaction. For SEM observations, surface of pristine Ti revealed relatively smooth and flat, while the surface morphology of coated Ti apparently changed as uniform and dense apatite layers were fully deposited on HA, L‐CHA, D‐CHA, and Rac‐CHA. Generally, the HA‐coated Ti showed a nanotopography composed of HA nanorods ≈100–500 nm length and nanoparticles ≈50–100 nm diameter. In comparison, the CHA coated Ti (L‐CHA, D‐CHA and Rac‐CHA) showed dense arrays of HA nanoplates ≈100–500 nm width and 10–60 nm thicknesses. In particular, as determined by samples in quadruplicate (Figure 2A; Figure S1, Supporting Information), SEM images of L‐CHA exhibited a stereospecific chiral hierarchical surface morphology, wherein left‐handed helical stacking of HA nanoplates could be observed at relatively low magnifications and higher magnified SEM images further indicated HA nanoplates were split into several HA nanoflakes ≈20–100 nm width. Consequently, the deposited apatite of L‐CHA formed a sheet‐like tertiary, secondary, and primary left‐handed chiral surface topography. In contrast, the right‐handed helical stacking of HA nanoplates observed in D‐CHA were further split into several HA nanoflakes ≈20–100 nm width, thus generating a sheet‐like tertiary, secondary, and primary right‐handed chiral surface topography. Additionally, the deposited nanoplates of Rac‐CHA were randomly arranged without stereospecific helical chiral structure detected. Of note, the left‐handed, right‐handed, and racemic helical arrangement of HA nanoflakes was visualized by the illustration below in L‐CHA, D‐CHA, and Rac‐CHA respectively (Figure 2A; Figure S1, Supporting Information).

The SEM images in Figure 2B revealed the CHA coating vertically deposited on Ti substrates and formed cluster‐like structures in L‐CHA, D‐CHA, and Rac‐CHA as compared to relatively smooth pristine Ti and HA at low magnifications. The EDS mapping of Ti, Al, Ca, P, and O elements further exhibited successful deposition of CaP crystals on Ti substrates and the distribution of coatings were basically homogeneous for each group. The crystalline structure of CHA‐coated Ti examined by XRD was shown in Figure 2C, in which the stronger intensity of the Ti substrates dominated the XRD patterns of samples and the characteristic reflections of hexagonal phase of HAP with the space group P63/m with lattice parameters of a = b = 9.4166 Å and c = 6.8745 Å (JCPDF No. 09‐0432) were indistinguishable. To address this issue, powders of CHA coatings were independently applied to XRD analysis as shown in Figure 2D, in which characteristic peaks (002 plan, 211 plan, 310 plan, 213 plan) for HA crystals well matched the Identification card (JCPDF No. 09‐0432) for each group, indicating main components of the coatings were HA.

The chirality of CHA‐coated Ti was definitely confirmed by optical activities (OAs) based on diffuse reflectance ultraviolet–visible (UV–vis) and circular dichroism (CD) spectroscopy as shown in Figure 2E. Of note, L‐CHA and D‐CHA exhibited antipodal CD signals, wherein L‐CHA exhibited two broad bands (200–350 nm and 350–800 nm) in the UV–vis spectrum and two negative shoulders (200–400 nm and 400–800 nm) and intense peaks (≈303 nm) in the CD spectrum, respectively. Consequently, the negative CD signal confirmed the left‐handed chirality of L‐CHA, while the opposite CD signal of D‐CHA reflected its right‐handed chirality. In comparison, the CD signal of Rac‐CHA revealed parallel to that of pristine Ti and HA without specific chirality. Consistently, the specific chirality of CHA powders was verified by their antipodal CD signals as shown in Figure S2 (Supporting Information). For surface hydrophilicity evaluation shown in Figure 2F, the average contact angle of pristine Ti (95.361°) were distinctly higher than that of HA (13.270°), L‐CHA (16.007°), D‐CHA (17.201°) and Rac‐CHA (15.725°), suggesting improved hydrophilicity of coated Ti with respect to pristine Ti. Additionally, the surface roughness quantified by AFM significantly elevated in CHA‐coated Ti (L‐CHA, D‐CHA, and Rac‐CHA) than pristine Ti and HA, and Rac‐CHA further increased surface roughness of samples as compared to L‐CHA and D‐CHA (Figure 2G,H).

In our previous researches, we have discovered that CD with white or black backgrounds revealed approximately solely absorption‐based OAs (AOAs) or scattering‐based OAs (SOAs) together with AOAs since all visible light is basically reflected by white light and absorbed by the black backboard, respectively.^[^ ^21^, ^22^ ^]^ Moreover, nanounits of the semiconductor aggregate in a chiral manner with distances shorter than the Bohr exciton radius known to induce a dissymmetric field on the entire aggregate through the delocalization of excitation, which results in AOAs based on electronic transition.^[^ ^21^, ^22^ ^]^ Consequently, the left‐handed structure prefers to reflect left‐handed light and absorbs right‐handed circularly polarized light, resulting in negative AOAs and SOAs signals for CD measurements. Based on this detection mechanism, the mirror‐imaged CD spectrum of L‐CHA and D‐CHA indicates their antipodal chiral topography as shown in Figure 2E, while pristine Ti, HA, and Rac‐CHA revealed no specific chirality and thus could be regarded as a black background, indicating OAs of samples arose from CHA coatings instead of Ti substrates. More importantly, the negative CD signal of L‐CHA undoubtedly confirms its highly enantioselective left‐handed chirality and vice versa for D‐CHA.

Therefore, the SEM‐EDS results visually confirmed CHA was successfully deposited on Ti substrates as indicated by the directional helical arrangement of HA nanoflakes, resulting in distinct stereospecific enantioselective topography shown in Figure 2A and Figure S1 (Supporting Information). Given the surfaces of CHA‐coated Ti show comparable microscale but considerable different nanoscale characteristics, the topography of samples was further examined by AFM. The significantly increased surface roughness of CHA‐coated Ti further confirmed the deposition of dense nano‐HA layers on Ti substrates. Moreover, based on the multi‐levels helical stacking of HA nanoplates induced by chiral self‐assembly, the roughness of CHA‐coated Ti revealed much higher than that of HA‐coated Ti which was characterized by relatively simple HA nanorods morphology, and the roughness of Rac‐CHA remarkably elevated probably due to its random arrangement of HA nanoplates without stereospecific chiral structure. On the other hand, nanotopography rough surfaces have been reported to increase surface energy and hydrophilicity of materials with respect to flat surfaces, which can strongly facilitate hydrophilic proteins adsorption such as fibronectin to regulate cell attachment.^[^ ^26^ ^]^ Consistently, our results also demonstrated the hydrophilicity of CHA‐coated Ti remarkably increased as compared to pristine Ti, which could probably be beneficial for samples to actively interact with biological systems.

### 2.2 In Vivo Implants Osseointegration Evaluation

Following 2 months bilateral ovariectomy, the bone density parameters (BV/TV and Tb.N) revealed significantly lower in OVX rats than Sham rats as analyzed by micro‐CT (Figure S3, Supporting Information), suggesting a successful establishment of OVX rats model. Moreover, the immunofluorescence staining of histological sections revealed distinctly higher NOS2 expressions in OVX rats than Sham rats (Figure S4A,B, Supporting Information), which manifested a relatively pro‐inflammatory condition generated by OVX rats. Meanwhile, the innervation and vascularization levels remarkably declined in OVX rats as indicated by their significantly decreased CGRP‐stained sensory nerves and CD31‐stained vessels (Figure S4A,C, Supporting Information).

**Figure 3:**
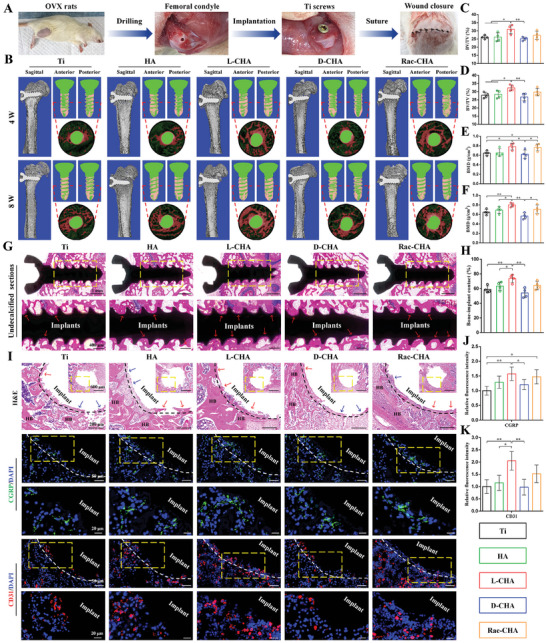
Evaluation of implants osseointegration by micro‐CT and histological analysis. A) Implantation procedures of materials into femoral condyle defects of OVX rats. B) Representative sagittal, anteroposterior axial, and median transverse images of implants reconstructed by 3D micro‐CT. The median transverse area was marked by red lines and magnified for observation (green for implanted Ti screws and red for bone formation). C–F) Quantitative analysis of micro‐CT data in terms of (C,D) BV/TV and (E,F) BMD. G,H) Undecalcified sagittal sections of samples and quantification of BIC at 8 weeks post‐implantation. I) Median transverse sections of samples stained by H&E (first row), CGRP (second and third row), and CD31 (fourth and fifth row) immunohistofluorescence at 4 weeks post‐implantation. J,K) Quantitative fluorescence intensity of (J) CGRP and (K) CD31. The areas marked by yellow box are magnified for observation; Black and white dashed lines indicate bone‐implant interface; Red arrows mark peri‐implant bone formation; Blue arrows mark fibrous tissues; HB represents host bone. *n* = 4. ^*^ *p* < 0.05, ^**^ *p* < 0.01.

The osseointegration of Ti screws after 4 and 8 weeks implantation were investigated by micro‐CT as shown in **Figure** **3**B, in which the bone formation (red) around implanted Ti screws (green) were 3D reconstructed and comprehensively exhibited from sagittal, anteroposterior axial and median transverse views. For quantitative analysis, BV/TV revealed significantly higher in L‐CHA than pristine Ti, HA, and D‐CHA following 4/8 weeks implantation, respectively (Figure 3C,D). Moreover, BMD were significantly higher in L‐CHA and Rac‐CHA than pristine Ti and D‐CHA at 4 weeks (Figure 3E), and with prolonged implantation period to 8 weeks, BMD were obviously higher in Rac‐CHA versus D‐CHA and remarkably higher in L‐CHA versus pristine Ti, HA, and D‐CHA (Figure 3F). Additionally, Tb.Th distinctly elevated in L‐CHA as compared to pristine Ti, D‐CHA, and Rac‐CHA at 4 weeks (Figure S5A, Supporting Information), and Tb.N were significantly higher in L‐CHA versus pristine Ti at 4 weeks (Figure S5B, Supporting Information). Moreover, Tb.Th revealed significantly higher in Rac‐CHA than D‐CHA and notably higher in L‐CHA than pristine Ti and D‐CHA at 8 weeks (Figure S5C, Supporting Information), while Tb.N significantly increased in L‐CHA as compared to pristine Ti, HA, and D‐CHA at 8 weeks (Figure S5D, Supporting Information).

For histological analysis, undecalcified sagittal sections were applied to explicitly exhibit BIC ratios as shown in Figure 3G, in which peri‐implant bone formation (red arrows) revealed much stronger in L‐CHA than other groups. On this basis, quantitative BIC ratios remarkably increased in L‐CHA than pristine Ti, HA, and D‐CHA following 8 weeks implantation (Figure 3H). Moreover, samples were decalcified and sectioned for H&E and immunohistofluorescence staining to comprehensively investigate peri‐implant immune reactions, innervation, and vascularization from median transverse views (Figure 3I; Figure S6, Supporting Information). The results indicated NOS2 expression levels significantly declined in Rac‐CHA than D‐CHA and notably declined in L‐CHA than pristine Ti and D‐CHA (Figure S6A,B, Supporting Information), while Arg‐1 expression levels distinctly elevated in L‐CHA and Rac‐CHA than pristine Ti and D‐CHA following 1 week implantation (Figure S6A,C, Supporting Information). Additionally, L‐CHA also greatly promoted Arg‐1 expression levels with respect to HA at 1 week post‐implantation (Figure S6A,C, Supporting Information). Moreover, L‐CHA obviously promoted peri‐implant bone formation (red arrows), accompanied by more CGRP^+^ (green) sensory nerves and CD31^+^ (red) endothelial cells ingrowth following 4 weeks implantation (Figure 3I). Consequently, quantitative CGRP expression levels significantly elevated in Rac‐CHA than pristine Ti and remarkably elevated in L‐CHA than pristine Ti and D‐CHA (Figure 3J), and L‐CHA yet notably promoted CD31 expressions than pristine Ti, HA and D‐CHA at 4 weeks post‐implantation (Figure 3K).

To investigate superior therapeutic potentials of CHA for bone healing, a rat femoral condyle defect model was applied to address osteoporotic osseointegration, since osteoporosis has become a highly prevalent bone disease that severely hinders osseointegration due to its overactive bone resorption coupled with hypoactive bone formation ability.^[^ ^27^ ^]^ In addition to osteoprogenitor cell lineages such as osteoblasts/osteoclasts, the immune, nervous, and vascular cells are all distributed adjacent to periosteum and bone trabeculae, thus generating a complicated interactive network to synergistically regulate bone metabolism and remodeling.^[^ ^28^ ^]^ Actually, pathological osteoporotic bone niches are not only caused by imbalance of coupled osteogenesis/osteoclastogenesis but also tightly associated with provocative chronic inflammation and impaired neurovascular distribution.^[^ ^28^, ^29^, ^30^ ^]^ Our results also indicated the deteriorated bone mass and microarchitecture accompanied by a provoked pro‐inflammatory state and dramatic reduction of innervation/vascularization densities in OVX rats. Under these circumstances, the osteoporotic osseointegration has become an urgent challenge in clinic, particularly abnormal biological fixation of orthopedic implants frequently happens and thus results in loosening, revision, or even failure of bone implantation.^[^ ^2^, ^3^, ^5^, ^31^ ^]^

Accordingly, strategies for bioinspired surface modification of orthopedic implants have been intensively explored to form strong bonds between bone‐implants interface.^[^ ^32^ ^]^ Among commercially available orthopedic implants, metal substrates, are most widely applied for providing immediate mechanical support and structural stability in forms of screws or plates.^[^ ^33^ ^]^ Thereinto, Ti alloys are preferred to be applied in orthopedics owing to their excellent biocompatibility, chemical stability, biomechanical properties, and improved bone‐implant contact abilities with respect to other metal implants such as zirconium and stainless steel.^[^ ^33^, ^34^ ^]^ However, the biological inertness and poor surfactivity of Ti remain a problem especially for repairing bone under pathological conditions. Thus, inorganic CaP coatings have emerged as a typical surface modification technique to fulfill higher clinical requirements of Ti implants.^[^ ^32^, ^35^ ^]^ In this view, Ti was selected as a substrate in this study and then modified by CHA coatings to generate stereospecific enantioselective interface for achieving ameliorative osteoporotic osseointegration.

Our in vivo results revealed L‐CHA coated Ti remarkably facilitated peri‐implant bone formation than other groups following 4 and 8 weeks implantation. Meanwhile, L‐CHA positively regulated the initial immune reactions from pro‐inflammatory to anti‐inflammatory states following 1 week implantation and induced much more sensory nerves and blood vessels ingrowth at 4 weeks post‐implantation. These results indicated L‐CHA induced remarkably strong osseointegration probably via manipulating initial immune responses coupled with subsequent innervation/vascularization around implants, which may balanced the local bone metabolism microenvironment under osteoporotic conditions. In contrast, Rac‐CHA revealed comparable peri‐implant bone formation, immunomodulation, innervation, and vascularization levels to pristine Ti and achiral HA, while D‐CHA showed the poorest biological performances. These results manifest enantioselective left‐handed chirality of L‐CHA dominated the bone‐implants interaction to potently promote osseointegration and vice versa for right‐handed chirality of D‐CHA, which synthetically resulted in a compromise outcome for racemic chirality of Rac‐CHA. In our previous researches, chirality‐induced biomaterials based on arginine, calcium silicate hydrate, and HA have been found to selectively favor soft (tendon) and hard (bone) tissues healing by a highly stereospecific L‐chirality rather than D‐/Rac‐chirality.^[^ ^19^, ^20^, ^21^, ^22^ ^]^ Consistently, recent studies also discovered L‐chiral engineered biomaterials possessed higher therapeutic potentials compared to their D‐chiral counterparts, mainly by ameliorating immune responses or promoting osteogenesis.^[^ ^23^, ^24^, ^36^, ^37^ ^]^ Taken together, the superior osseointegration ability of L‐CHA observed in the present study exclusively depended on its stereospecific left‐handed chiral hierarchical structure without doping of chemical or bioactive agents, thus may providing a more simple and practical strategy to meet the urgent clinical need for osteoporotic osseointegration.

### 2.3 Macrophage Responses to CHA‐Coated Ti

**Figure 4:**
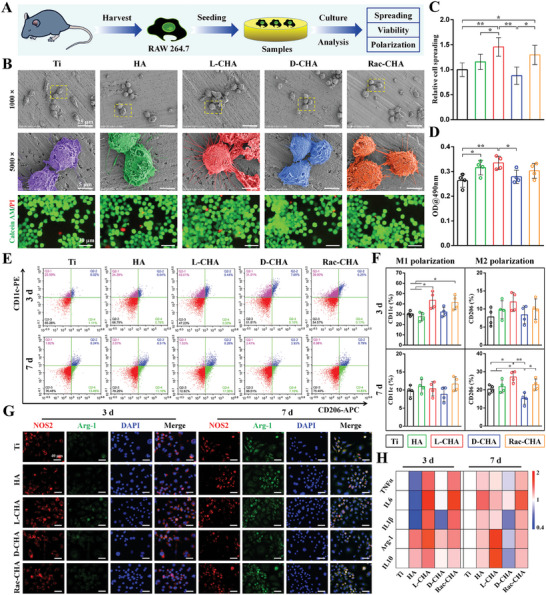
Macrophage responses to CHA‐coated Ti samples. A) Procedures of cell seeding on samples and biological examinations. B) RAW cells adhesion morphology and viability detected by SEM (first and second row) and live/dead staining (third row) respectively. The areas marked by yellow box are magnified and rendered for observation. C) RAW cells spreading quantified by aspect ratios. (D) RAW cells viability determined by MTT. E,F) RAW cells polarization quantified by CD11c (M1) and CD206 (M2) expressions using flow cytometry. G) RAW cells polarization evaluated by co‐staining of NOS2/Arg‐1 (M1/M2) immunofluorescence. H) Heatmap of pro‐inflammatory (TNFα, IL6, IL1β) and anti‐inflammatory (Arg‐1, IL10) gene expressions of RAW cells determined by RT‐PCR. *n* = 4. ^*^ *p* < 0.05, ^**^ *p* < 0.01.

As shown in **Figure** **4**B, abundant filopodia of RAW cells fully stretched on L‐CHA, while limited filopodia were observed on Rac‐CHA and HA. Moreover, the filopodia formation visibly decreased on pristine Ti and D‐CHA. Consequently, the cell spreading was distinctly promoted in Rac‐CHA than pristine Ti and D‐CHA, and L‐CHA further facilitated RAW cells spreading to a great extent as compared to Ti, HA, and D‐CHA (Figure 4C). For macrophage viability evaluation (Figure 4B), RAW cells grew well with few dead cells when cultured 3 days, revealing the satisfied biocompatibility of CHA coating and pristine Ti for macrophages. Moreover, significantly more viable RAW cells were detected in L‐CHA than other groups. The MTT assays further confirmed the enhanced ability of CHA coating for supporting RAW cells developments following 3 days culture, particularly L‐CHA remarkably promote RAW cells growth as compared to pristine Ti and RAW cells viability significantly elevated in HA and L‐CHA than pristine Ti and D‐CHA, respectively (Figure 4D).

As determined by flow cytometry assays, RAW cells treated by IFNγ and LPS revealed extremely higher CD11c levels than blank control (Figure S7, Supporting Information), suggesting the successful M1 phenotype induction of RAW cells. Following 3 days culture on each sample, M1 polarization was significantly up‐regulated in L‐CHA and Rac‐CHA than Ti and HA, while M2 polarization obviously elevated in Ti, HA, and Rac‐CHA with respect to D‐CHA after 7 days culture (Figure 4E,F). Of note, M2 polarization induced by L‐CHA was significantly higher than that of Ti and much more potent than that of D‐CHA for 7 days culture (Figure 4E,F).

The expression of NOS2/Arg‐1 (M1/M2) detected by immunofluorescence exhibited a similar tendency to the flow cytometry analysis. As shown in Figure 4G and Figure S8 (Supporting Information), the fluorescence intensity of NOS2 (M1 marker) was significantly stronger in L‐CHA versus Ti and HA on day 3, while the Arg‐1 (M2 marker) expression levels were significantly up‐regulated in L‐CHA and Rac‐CHA versus HA and D‐CHA respectively on day 7. Moreover, L‐CHA promoted Arg‐1 expressions to a great extent as compared to D‐CHA for 7 days culture (Figure 4G; Figure S8B, Supporting Information). Consistently, the pro‐inflammatory (TNFα, IL6, IL1β) and anti‐inflammatory (Arg‐1, IL10) gene expressions of RAW cells determined by RT‐PCR (Figure 4H) were respectively parallel to M1 and M2 polarization levels described above.

The initial in vivo bone‐implants interaction is predominately mediated by acute immunological reactions mainly driven by macrophages, which immediately recognize the implant as a foreign body and attach to implant's surface to shift polarization phenotypes (M1/M2) based on the surrounding milieu, thereby creating a crucial early immune microenvironment following implantation.^[^ ^38^ ^]^ Herein, to better replicate the in vivo early immune reactions state, RAW cells were directly seeded on samples and subsequently provoked by IFNγ and LPS to shift toward a pro‐inflammatory M1 polarization state. All samples well supported RAW cells growth and functionality without obvious cytotoxicity, suggesting the satisfied biocompatibility of samples.

Of note, L‐CHA notably facilitated RAW cells spreading and proliferation, accompanied by a significantly enhanced immunomodulation ability to propel RAW cells transition from pro‐inflammatory M1 to anti‐inflammatory M2 phenotypes. Given cellular behaviors are primarily affected by surface properties of materials, the positive immunomodulatory actions of L‐CHA could be partly attributed to its improved hydrophilicity and nanotopography rough surfaces, since the hydrophilic and rough surfaces modified by nano‐HA coatings are demonstrated able to favor the adhesion, viability and M1‐M2 phenotypes transition of RAW cells.^[^ ^25^, ^39^ ^]^ However, D‐CHA revealed opposite bioeffects to hinder RAW cells attachment, growth, and M1‐M2 polarization with respect to L‐CHA, despite it possessed comparable surface roughness and hydrophilicity to L‐CHA. This phenomenon suggests the fate of RAW cells was predominately governed by the stereospecific chiral hierarchical topography rather than surface roughness or hydrophilicity of samples, which further manifests chirality is a fundamental attribute of life science and hence plays critical roles to manipulate cellular immune responses.^[^ ^8^, ^9^, ^10^ ^]^

Actually, our previous researches have found the adhesion and viability of TSPCs and BMSCs were significantly promoted by chiral‐engineered membranes with stereospecific L‐chirality rather than D‐/Rac‐chirality.^[^ ^19^, ^21^ ^]^ On the other hand, recent studies have reported L‐chiral lactate tends to activate M2 macrophages, while D‐chiral amino acids prefer to stimulate M1 phenotype polarization.^[^ ^40^, ^41^ ^]^ Consistently, the present study also manifests the enantiomer‐dependent macrophage responses to CHA, wherein L‐CHA strongly facilitated RAW cells developments and M1‐M2 phenotypes transition, but D‐CHA could hardly favor the bioactivity of RAW cells. It is well acknowledged that pro‐inflammatory M1 macrophages dominate the early implantation stage to initiate neovascularization and cell chemotaxis, while anti‐inflammatory M2 macrophages are principally responsible for tissue regeneration during later implantation periods.^[^ ^5^, ^25^ ^]^ That's to say, either M1 or M2 macrophage is essential for tissue healing and the implantation outcomes are determined by the macrophage polarization switching pattern rather than one specific M1/M2 phenotype.^[^ ^38^, ^42^ ^]^ Therefore, the present study highlights the desired immunomodulation ability of L‐CHA to induce efficient and timely macrophages M1‐M2 phenotype transition mainly relying on the stereospecific left‐handed chiral hierarchical topography along with improved hydrophilicity and nanotopography rough surfaces, which could provide a positive early immune microenvironment for subsequent bone regeneration process.

### 2.4 Activation of DRG/HUVECs Cultured on Samples

**Figure 5:**
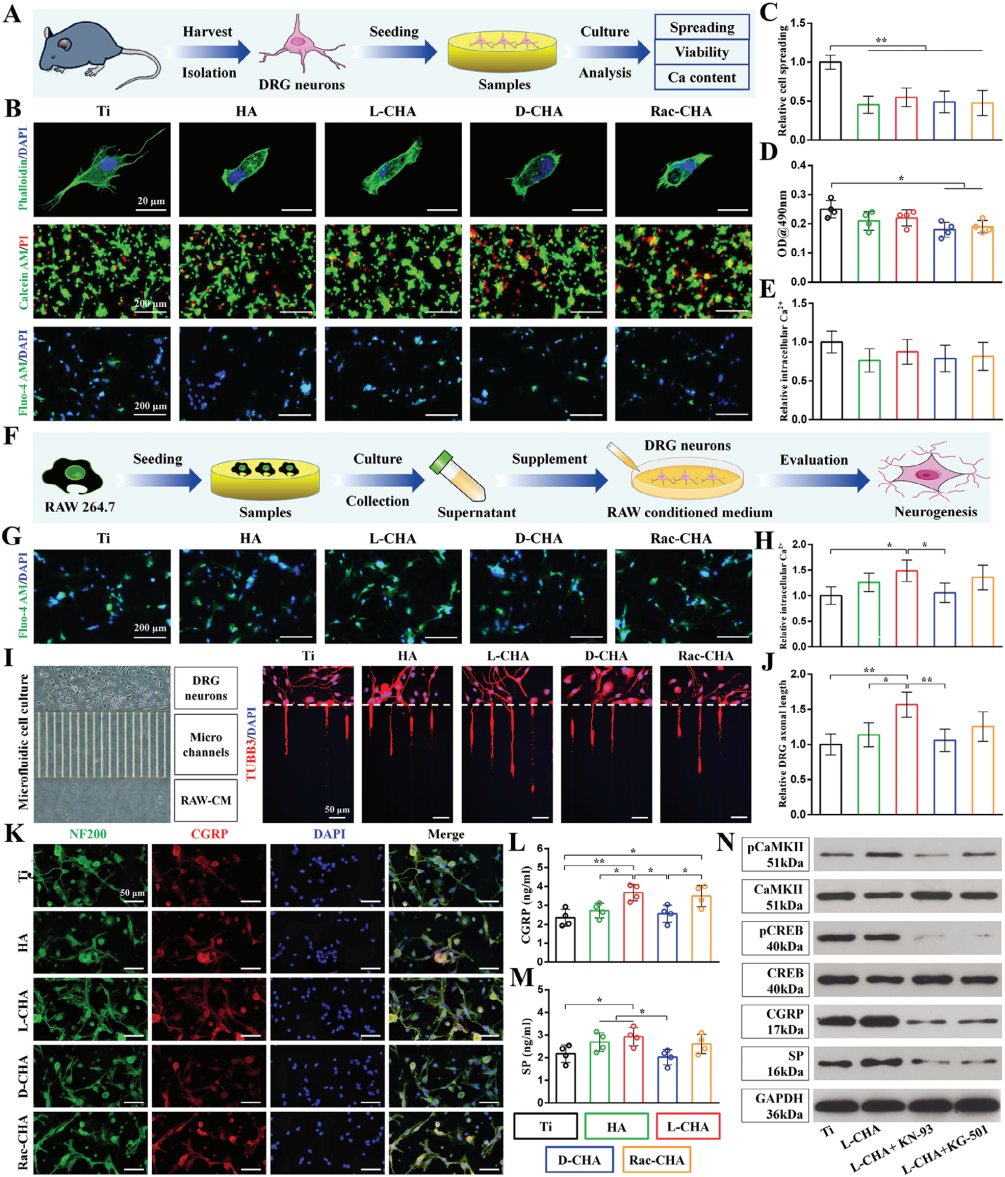
Cellular activities of DRG neurons cultured on CHA coated Ti and the immuno‐inductive neurogenesis evaluation of samples. A) Procedures of cell seeding on samples and biological examinations. B) The adhesion morphology, viability, and bioactivity of DRG neurons detected by cell actin cytoskeleton staining (first row), live/dead staining (second row), and Ca^2+^ fluorescence probe (third row) respectively. C) Cell spreading quantified by aspect ratios. D) Viability of DRG neurons determined by MTT. E) Quantification of intracellular Ca^2+^ based on fluorescence intensity of Ca^2+^ probe. F) In vitro immuno‐inductive neurogenesis of DRG neurons stimulated by RAW conditioned medium. G,H) Immuno‐inductive intracellular Ca^2+^ expressions examined by Ca^2+^ fluorescence probe. I,J) Immuno‐inductive DRG neurons axonal growth determined by microfluidics assays. White dashed lines indicate microgroove barriers (500 µm) of the microfluidic device. K) Immuno‐inductive CGRP and NF200 expressions detected by immunofluorescence co‐staining. L,M) Immuno‐inductive (L) CGRP and (M) SP expressions quantified by ELISA. N) Western blot analysis of immuno‐inductive CGRP and SP expressions in L‐CHA supplemented with/without KN‐93 or KG‐501. *n* = 4. ^*^ *p* < 0.05, ^**^ *p* < 0.01.

DRG neurons exhibited specific cytoskeletal orientation and functional axonal autogrowth on pristine Ti but showed insufficient axonal elongation and unspecific spherical shape when cultured on CHA‐coated Ti for 1 day (**Figure** **5**B). Consequently, the quantitative aspect ratios remarkably decreased in CHA‐coated Ti as compared to pristine Ti (Figure 5C), suggesting the hampering effects of CHA coating for DRG neurons spreading. Moreover, as compared to pristine Ti, the viability of DRG neurons cultured for 3 days declined in CHA‐coated Ti especially for D‐CHA and Rac‐CHA, in which fewer viable DRG neurons (green) accompanied by increased amounts of dead cells (red) were detected (Figure 5B). Accordingly, the MTT assays demonstrated the growth of DRG neurons were significantly inhibited in D‐CHA and Rac‐CHA versus pristine Ti (Figure 5D). Additionally, a declined tendency of intracellular Ca^2+^ levels was observed in DRG neurons cultured for 3 days on CHA‐coated Ti versus pristine Ti (Figure 5B,E).

**Figure 6:**
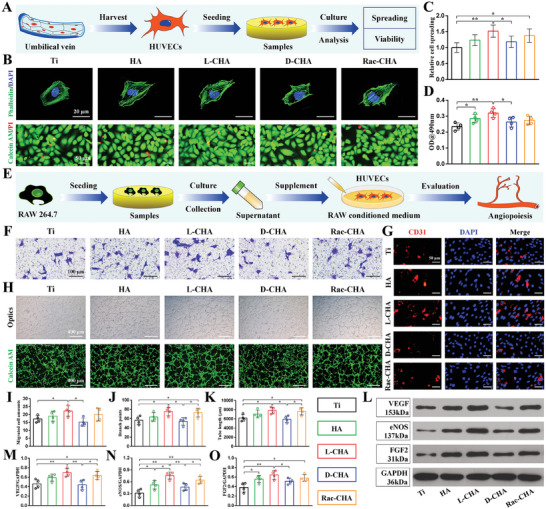
Cellular activities of HUVECs cultured on CHA‐coated Ti and the immuno‐inductive angiogenesis evaluation of samples. A) Procedures of cell seeding on samples and biological examinations. B) The adhesion morphology and viability of HUVECs detected by cell actin cytoskeleton staining (first row) and live/dead staining (second row) respectively. C) Cell spreading quantified by aspect ratios. D) Viability of HUVECs determined by MTT. E) In vitro immuno‐inductive angiogenesis of HUVECs stimulated by RAW‐conditioned medium. F,I) Immuno‐inductive migration of HUVECs examined by transwell assays. G) Immuno‐inductive CD31 expressions detected by immunofluorescence. H) Immuno‐inductive tube formation detected via ordinary microscope (first row) and calcein AM fluorescence (second row). J,K) Quantification of (J) branch points number and (K) tube length based on tube formation images. L) Immuno‐inductive angiogenesis‐related proteins (VEGF, eNOS, FGF2) expressions detected by western blot. M–O) Quantification of (M) VEGF, (N) eNOS, and (O) FGF2 expressions based on western blot results. *n* = 4. ^*^ *p* < 0.05, ^**^ *p* < 0.01.

For the evaluation of HUVECs adhesion after 1 day culture, actin cytoskeleton stained by phalloidin revealed a relatively round shape of HUVCEs cultured on pristine Ti and visibly stretched cell morphology cultured on CHA coated Ti especially for HA, L‐CHA, and Rac‐CHA (**Figure** **6**B). Therefore, the cell spreading was significantly improved in HA and Rac‐CHA versus pristine Ti, and particularly L‐CHA promoted the spreading of HUVECs to a greatest level, which was significantly higher than that of D‐CHA and notably higher than that of pristine Ti (Figure 6C). Following 3 days culture, HUVECs grew quite well without obvious dead cells (red) detected on each group of samples, suggesting the excellent biocompatibility of CHA coating and pristine Ti for endothelial cells (Figure 6B). Additionally, the MTT assays indicated improved viability of HUVECs cultured on CHA‐coated Ti, wherein HA and L‐CHA significantly promote HUVECs growth with respect to pristine Ti and D‐CHA respectively, and L‐CHA further greatly promote HUVECs growth as compared to pristine Ti (Figure 6D).

In the present study, DRG neurons and HUVECs were directly cultured on samples to investigate the neurogenesis/angiogenesis potentials of CHA. Interestingly, our results indicate the cellular developments of DRG neurons stimulated by CHA were far from those of HUVECs, in which CHA‐coated Ti obviously promoted the cell attachment and viability of HUVECs but hindered the spreading and bioactivity of DRG neurons.

Considering cell adhesion is the initial step to determine cellular interactions with materials, the disadvantageous bioeffects of CHA‐coated Ti on DRG neurons are probably caused by their improved hydrophilicity and nanotopography rough surfaces, which are likely to block the adsorption of important hydrophobic neural adhesive proteins (e.g., laminin) and thus impede the initial sensing process mediated by focal adhesion complexes of neurons.^[^ ^43^ ^]^ As previously reported, neurons fully spread and grow well on flat substrates, while neurons cultured on rough substrates tend to aggregate for their functionally impaired actin cytoskeleton.^[^ ^43^, ^44^ ^]^ Our recent research also discovered the viability of neural SCs was significantly decreased on chiral MoS_2_ nanoplates coated bacterial cellulose (BC) membranes versus smooth BC substrates.^[^ ^45^ ^]^ Therefore, the cellular spreading, viability, and bioactivity of DRG neurons obviously declined as surface roughness increased from flat pristine Ti to nanoscaled rough CHA‐coated Ti, despite the stereospecific chiral topography may positively activate neural cells under certain circumstances.^[^ ^45^, ^46^ ^]^ In comparison, the hydrophilicity and nanotopography rough surfaces are reported quite beneficial for cellular activities of HUVECs^[^ ^25^, ^26^ ^]^ and the stereospecific L‐chiral hierarchical structures can also favor angiogenesis of endothelial cells,^[^ ^20^ ^]^ thereby accounting for the considerable bioeffects of L‐CHA to directly promote bioactivity of HUVECs.

### 2.5 Neurogenesis/Angiogenesis Stimulated by RAW‐CM

Intracellular Ca^2+^ in DRG neurons stimulated by RAW‐CM for 3 days significantly raised in L‐CHA as compared to pristine Ti and D‐CHA (Figure 5G,H). For evaluation of immuno‐inductive DRG neurons axonal growth, axons of DRG neurons could protruded into microchannels of a microfluidic device and the neurite outgrowth were visibly promoted by RAW‐CM of L‐CHA following 3 days incubation (Figure 5I). Consequently, quantitative DRG axonal length remarkably elevated in L‐CHA with respect to pristine Ti, HA, and D‐CHA (Figure 5J). Meanwhile, the fluorescence intensity of sensory neuropeptides CGRP revealed obviously stronger in Rac‐CHA versus D‐CHA and distinctly stronger in L‐CHA than pristine Ti and D‐CHA following 3 days culture in RAW‐CM (Figure 5K; Figure S10, Supporting Information), while the fluorescent SP expressions were significantly higher in HA versus D‐CHA and remarkably up‐regulated in L‐CHA than pristine Ti and D‐CHA (Figures S9 and S10, Supporting Information). Consistently, ELISA assays further indicated CGRP concentrations in DRG neurons notably elevated in L‐CHA than pristine Ti, HA, and D‐CHA, while Rac‐CHA yet significantly facilitated CGRP secretion as compared to pristine Ti and D‐CHA after 3 days culture in RAW‐CM (Figure 5L). In addition, SP concentrations in DRG neurons revealed obviously higher in HA versus D‐CHA and significantly higher in L‐CHA than pristine Ti and D‐CHA following 3 days culture in RAW‐CM (Figure 5M).

Moreover, to investigate the potential pathway for immuno‐inductive neurogenesis of L‐CHA, we supplied KN‐93 (CaMKII inhibitor) or KG‐501 (CREB inhibitor) to RAW‐CM of L‐CHA for DRG neurons culture. Consequently, after 3 days culture in RAW‐CM, CGRP and SP proteins expressions of DRG neurons were significantly up‐regulated in L‐CHA as compared to pristine T (Figure 5N; Figure S11C,D, Supporting Information), accompanied by the enhanced activation of pCaMKII and pCREB (Figure 5N; Figure S11A,B, Supporting Information). In comparison, the supplement of either KN‐93 or KG‐501 to RAW‐CM of L‐CHA notably inhibited the activation of pCaMKII and pCREB (Figure 5N; Figure S11A,B, Supporting Information), resulting in considerable reduction of CGRP and SP expressions in DRG neurons cultured for 3 days (Figure 5N; Figure S11C,D, Supporting Information). In particular, the supplement of KN‐93 remarkably inhibited the phosphorylation of both CaMKII and CREB, while KG‐501 significantly impeded the phosphorylation of CREB but just slightly inhibited the activation of pCaMKII for DRG neurons (Figure S11A,B, Supporting Information), suggesting CREB is probably a downstream effector of CaMKII. Taken together, these results indicated that RAW‐CM of L‐CHA up‐regulated CGRP and SP expressions of DRG neurons probably by activating the CaMKII/CREB signaling pathway.

After stimulated by RAW‐CM for 12 h, larger amounts of HUVECs migrated across transwell plates in L‐CHA than pristine Ti and D‐CHA (Figure 6F,I). Consistently, CD31 expressed by HUVECs significantly elevated in L‐CHA than pristine Ti and D‐CHA following 3 days culture in RAW‐CM (Figure 6G; Figure S12, Supporting Information). For evaluation of tube formation incubated in RAW‐CM for 4 h, the branch points and tube length of HUVECs distinctly increased in L‐CHA and Rac‐CHA versus pristine Ti and D‐CHA (Figure 6H,J,K). Moreover, the western blot results further confirmed the enhanced immuno‐inductive angiogenesis ability of CHA‐coated Ti, particularly L‐CHA notably up‐regulated VEGF, eNOS and FGF2 proteins expressions of HUVECs as compared to pristine Ti and D‐CHA after 3 days culture in RAW‐CM (Figure 6L–O). In comparison, Rac‐CHA remarkably facilitated VEGF and eNOS proteins expressions of HUVECs with respect to pristine Ti and D‐CHA following 3 days culture in RAW‐CM (Figure 6L–N). Additionally, eNOS and FGF2 proteins expressions significantly elevated in HA than pristine Ti (Figure 6L,N,O), while L‐CHA also distinctly promoted eNOS proteins expressions as compared to HA (Figure 6L,N), and Rac‐CHA significantly promoted FGF2 proteins expressions with respect to pristine Ti (Figure 6L,O).

To better understand the bioeffects of CHA on dictating bone‐implants interaction, the immuno‐inductive neurogenesis/angiogenesis were further investigated since bone healing is a synergetic and chronological process initiated by early immune reactions accompanied by neurovascularization.^[^ ^28^, ^29^, ^38^ ^]^ Recently, the focus of bone research has shifted from conventional angiogenesis/osteogenesis to immune/neural systems mainly for the acknowledged immunomodulation and skeletal interoception functions, which are highlighted to act as pioneers for manipulating the initial period of bone healing.^[^ ^38^, ^47^, ^48^ ^]^ On this basis, the neuro‐immune interactions have emerged as a hot biomedical frontier,^[^ ^49^ ^]^ particularly a sophisticated immune‐neural signaling axis indicates upstream macrophages triggered by implants can produce inflammatory molecules (e.g., PGE2) to activate DRG sensory nerves via the CREB signaling and thus promote bone formation.^[^ ^50^ ^]^

Likewise, our results also revealed L‐CHA significantly stimulated the bioactivity, axon growth, and neurogenesis of DRG neurons probably by activating the CaMKII/CREB signaling pathway of macrophages. The varied performances of DRG neurons stimulated by RAW‐CM with respect to direct culture on samples indicate a potent mediator role of macrophages and, therefore, the enhanced immuno‐inductive neurogenesis of L‐CHA could be rationally responsible for their accelerated peri‐implant innervation observed in vivo, despite L‐CHA appeared unable to directly favor the neurogenesis of DRG neurons. Parallelly, L‐CHA strongly facilitated the migration, tube formation, and angiogenic activities of HUVECs when stimulated by RAW‐CM, thus validating the dual immuno‐inductive actions of L‐CHA to promote neurogenesis and angiogenesis. Overall, these results suggest the satisfied osteoporotic osseointegration ability of L‐CHA was probably depending on its well‐orchestrated immunomodulation coupled with neurogenesis and angiogenesis.

### 2.6 Osteogenesis Stimulated by CHA‐Coated Ti

**Figure 7:**
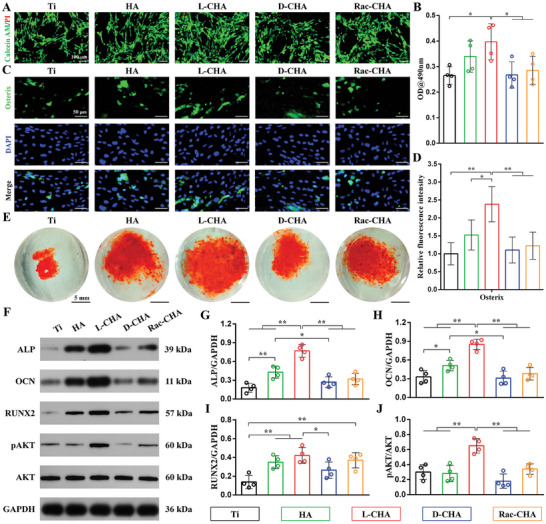
Osteogenesis of BMSCs cultured on CHA‐coated Ti. A) Cell viability detected by live/dead staining for 3 days culture. B) Cell viability determined by MTT assays. C,D) Fluorescent quantitation of osterix expressions. E) ARS staining for 14 days culture. F) Osteogenesis‐related proteins (ALP, OCN, RUNX2, pAKT) expressions analyzed by western blot. G–J) Quantification of (G) ALP, (H) OCN, (I) RUNX2, and (J) pAKT expressions based on western blot results. *n* = 4. ^*^ *p* < 0.05, ^**^ *p* < 0.01.

As shown in **Figure** **7**A, BMSCs grew quite well without obvious dead cells detected in each group, thus further validating the excellent biocompatibility of samples. The quantitative MTT assays indicated viability of BMSCs significantly elevated in L‐CHA than pristine Ti, D‐CHA, and Rac‐CHA (Figure 7B). For osteogenic differentiation assessment, the fluorescent osterix expression revealed remarkably stronger in L‐CHA than other groups for 3 days culture (Figure 7C,D). The ARS staining on day 14 also reflected the notably increased mineralization nodules induced by CHA‐coated Ti especially for L‐CHA (Figure 7E). Moreover, the western results revealed L‐CHA remarkably promoted osteogenic markers (ALP, OCN) expressions than other groups, and HA yet facilitated ALP and OCN protein expressions than pristine Ti and D‐CHA for 7 days culture (Figure 7F–H). Additionally, RUNX2 expression levels were significantly higher in HA, L‐CHA, and Rac‐CHA than pristine Ti, and L‐CHA further promoted RUNX2 expressions than D‐CHA for 7 days culture (Figure 7F,I). In particular, the phosphorylation level of AKT (pAKT) was detected notably up‐regulated in L‐CHA than other groups (Figure 7F,J).

In view of this, the AKT phosphorylation inhibitor (MK2206) was subsequently applied to investigate whether the withdrawal of pAKT could affect osteogenesis effects of L‐CHA. As revealed, L‐CHA significantly promoted OCN and RUNX2 protein expressions than pristine Ti for 14 days culture (Figure S13A,C,D, Supporting Information), accompanied by distinctly elevated pAKT activation levels (Figure S13A,E, Supporting Information). However, the supplement of MK2206 compulsively blocked AKT phosphorylation and thus greatly impeded the activation of pAKT (Figure S13A,E, Supporting Information), resulting in dramatic down‐regulation of osteogenic markers (ALP, OCN, RUNX2) expressions in L‐CHA (Figure S13A–D, Supporting Information).

The results confirm the superior osteogenesis effects of L‐CHA to promote viability, osteogenic differentiation, and mineralization of BMSCs than pristine Ti and other CHAs, which are consistent to our previous research that hierarchical L‐chiral HA possessed desired stereospecific anisotropy to selectively favor osteogenic differentiation of MSCs.^[^ ^21^ ^]^ In spite of the positive enantioselective interactions between L‐chirality and stem cells, the related signaling mechanism is rarely investigated and remains poorly understood. Herein, to our best knowledge, it is the first time we explored the key signaling molecule accounting for enhanced osteogenesis ability of L‐chiral HA. Consequently, the expression levels of osteogenic markers (ALP, OCN, RUNX2) in L‐CHA revealed highly positive correlation with phosphorylation levels of AKT (pAKT), wherein the activation/blocking of pAKT strongly promoted/inhibited osteogenic differentiation of BMSCs. As known, pAKT is a key molecule participating in the activation of PI3K/AKT signaling pathway which is tightly linked to osteogenic activities, and recent study has reported hierarchical micro‐nano morphology notably up‐regulated PI3K/AKT pathway to facilitate osteogenic differentiation.^[^ ^51^ ^]^ Consistently, our results preliminarily demonstrated the considerable roles of PI3K/AKT signaling on promoting osteogenesis of L‐CHA, which may provide a new perspective for better understanding the enantiomer‐dependent osteogenesis bioactivity.

### 2.7 Immunomodulation Mechanism of Samples

**Figure 8:**
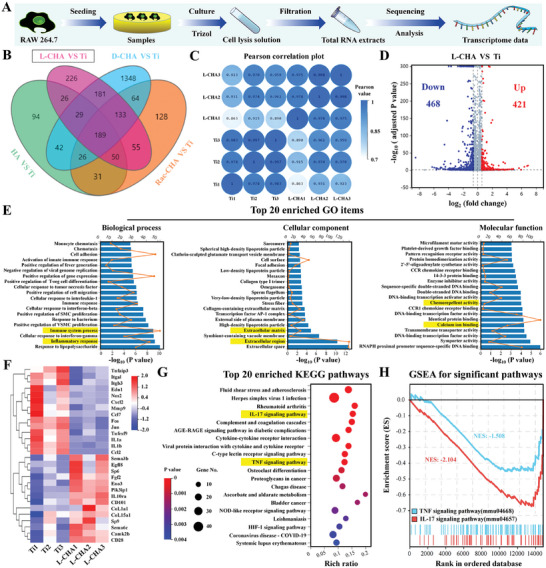
Mechanism of samples on modulating macrophage reactions analyzed by transcriptomic RNA‐seq. A) Procedures of RAW cells culture and subsequent RNA‐seq analysis. B) Venn diagram of differentially expressed genes (DEGs) amounts between CHA‐coated Ti and pristine Ti. C) Pearson correlation plot of L‐CHA and pristine Ti samples. D) Volcano plot of DEGs in L‐CHA versus pristine Ti. E) GO analysis of DEGs in L‐CHA versus pristine Ti. F) Heatmap of partial DEGs mainly involved in inflammation, angiogenesis, and neurogenesis. G) Top 20 enriched KEGG pathways based on DEGs of L‐CHA versus pristine Ti. H) GSEA analysis for significant pathways (IL‐17 and TNF signaling pathway) down‐regulated by L‐CHA versus pristine Ti. The yellow marks highlight interested GO items and KEGG pathways.

To clarify the underlying mechanism how samples modulated macrophage activities, transcriptomic analysis of RAW cells cultured on each groups of samples was conducted. As shown in Venn diagram (**Figure** **8**B), differentially expressed genes were comprehensively analyzed in CHA‐coated Ti as compared to pristine Ti (487 genes in HA versus pristine Ti, 889 genes in L‐CHA versus pristine Ti, 2012 genes in D‐CHA versus pristine Ti and 676 genes in Rac‐CHA versus pristine Ti). Particularly, the positive group L‐CHA were individually compared to the control group pristine Ti, and the distribution of three samples in L‐CHA revealed quite apart from those in pristine Ti as determined by PCA analysis (Figure S14, Supporting Information), indicating the whole gene expression distinctly varied between L‐CHA and pristine Ti. Additionally, the Pearson correlation plot demonstrated the satisfied stability of RNA samples obtained from L‐CHA and pristine Ti, as indicated by the high Pearson value (>0.97) between samples in each group (Figure 8C).

The volcano plot further clarified 421 up‐regulated genes and 468 down‐regulated genes in L‐CHA versus pristine Ti (Figure 8D). Interested genes involved in anti‐inflammation (IL10ra, CD101), angiogenesis (Fgf2, Egfl8, Eno3), neurogenesis (Sema3b, Sema6c) and osteogenesis (Camk2b, Pik3ipl, CoL1a1, CoL15a1) were notably up‐regulated in L‐CHA, while genes related to pro‐inflammation (Tnfaip3, Nos2, IL1a, IL1b, Tnfrsf9, Fos, Jun), cell chemotaxis (Cxcl2, Ccl7, Ccl2, Mmp9), cell binding (Itgal, Itgb3) and vasoconstriction (Edn1) were significantly down‐regulated in L‐CHA with respect to pristine Ti (Figure 8F). Notably, top 20 enriched GO items based on DEGs between L‐CHA and pristine Ti were comprehensively analyzed and, therefore, the biological process, cellular component, and molecular function were found specifically enriched in Inflammatory response, Immune system process, Extracellular region, Extracellular matrix, Calcium ion binding, Chemorepellent activity, etc. as shown in Figure 8E. Moreover, the enriched KEGG pathways and GSEA analysis confirmed the down‐regulated IL‐17 signaling pathway was principally responsible for the immunomodulatory actions of L‐CHA as compared to pristine Ti (Figure 8G,H). Additionally, TNF signaling pathway was also down‐regulated in L‐CHA, suggesting L‐CHA predominately down‐regulated IL‐17 signaling pathway along with TNF signaling pathway to modulate M1‐M2 macrophage transition as compared to pristine Ti (Figure 8G,H).

Likewise, the RNA‐seq analysis further indicated there were 14 891 overlapped genes between L‐CHA and D‐CHA, along with 761 genes specifically expressed by L‐CHA and 656 genes specifically expressed by D‐CHA (Figure S15A, Supporting Information). The volcano plot clarified 483 up‐regulated genes and 438 down‐regulated genes in L‐CHA versus D‐CHA (Figure S15B, Supporting Information). On this basis, the enriched KEGG pathways and GSEA analysis confirmed the down‐regulated Osteoclast differentiation and IL‐17 signaling pathway could probably account for the positive immunomodulatory actions of L‐CHA as compared to D‐CHA (Figure S15C,D, Supporting Information).

In light of the above biological results, the outstanding immunomodulation action of L‐CHA was considered as a crucial prerequisite to promote bone healing. The RNA‐seq analysis validated the positive immunomodulatory actions of L‐CHA to manipulate desired M1‐M2 macrophage transition coupled with enhanced innervation, vascularization, and osseointegration, as indicated by the up‐regulated genes related to anti‐inflammation (IL10ra, CD101), angiogenesis (Fgf2, Egfl8, Eno3), neurogenesis (Sema3b, Sema6c), osteogenesis (Camk2b, Pik3ipl, CoL1a1, CoL15a1) along with down‐regulated pro‐inflammation (Tnfaip3, Nos2, IL1a, IL1b, Tnfrsf9, Fos, Jun) genes. Under comprehensive analysis between L‐CHA and pristine Ti/D‐CHA, the enriched KEGG pathways and GSEA analysis clarified the desired immunomodulation ability of L‐CHA may be predominately attributed to the concurrently down‐regulated IL‐17 signaling pathway.

Thus, the underlying signal transduction process was further explored based on the PPI network generated by RNA‐seq, which displayed key genes involved in the down‐regulated IL‐17 signaling pathway of L‐CHA versus pristine Ti (Figure S16A, Supporting Information). Then, the acknowledged IL17 molecular family including IL‐17A, IL‐17F, IL‐17B, and IL‐17C was analyzed by RT‐PCR, which indicated L‐CHA significantly down‐regulated IL‐17C gene expressions with respect to pristine Ti (Figure S16B, Supporting Information). Moreover, the western results also revealed IL‐17C protein expressions were remarkably down‐regulated in L‐CHA than pristine Ti, HA, and D‐CHA (Figure S16C,D, Supporting Information), accompanied by significant reduction of pNF‐κB protein expressions in L‐CHA than pristine Ti and D‐CHA (Figure S16C,F, Supporting Information). Additionally, Mmp9 protein expressions were yet significantly down‐regulated in L‐CHA than pristine Ti (Figure S16C,G, Supporting Information). These results were basically coincident with the PPI network generated by RNA‐seq, thereby suggesting the down‐regulation of IL‐17 signaling pathway in L‐CHA was probably mediated by inhibiting IL‐17C/NF‐κB signaling axis (Figure S17, Supporting Information) along with decreasing Mmp9 production as previously reported.^[^ ^52^ ^]^

It is acknowledged that IL‐17 is a extremely versatile pro‐inflammatory cytokine participating in multiple biological processes including protective immune responses, inflammatory cascade defenses, cancer progression and tissue repair.^[^ ^53^ ^]^ Recently, the multifunctional IL‐17 signaling pathway has gained intensive attention mainly owing to its great impacts on organism immunoregulation.^[^ ^54^ ^]^ Regardless, the biological function and transduction approaches of IL‐17 signaling pathway remain controversial in many cases and,^[^ ^54^ ^]^ therefore, it's the first time we discovered the down‐regulated IL‐17 signaling pathway may be principally responsible for L‐CHA to exert its potent immunomodulatory actions for bone healing. Indeed, previous researches have reported down‐regulation of IL‐17 signaling pathway is able to suppress the activation of pro‐inflammatory macrophages and thus ameliorate inflammation‐induced bone loss, showing great potentials for treating rheumatoid arthritis complicated with osteoporosis.^[^ ^55^ ^]^ On the other hand, the down‐regulated TNF signaling pathway frequently occurs in accompany with down‐regulated IL‐17 signaling pathway to conjointly attenuate hyperactive inflammation,^[^ ^56^, ^57^ ^]^ which coincides with the results observed in the present study. In summary, the transcriptomic RNA‐seq analysis rationally explains the desired biological functionality of L‐CHA, and the newly discovered down‐regulated IL‐17 signaling pathway in L‐CHA may provide a theoretical basis for better understanding the mechanism underlying L‐chirality's roles for bone healing.

Nevertheless, there are still several challenges with present study to be addressed. First, how to precisely control the chiral induction process and achieve higher specificity, stability, and reproducibility of CHA. On the other hand, the in‐depth signaling and molecular mechanism of CHA on dictating bone healing needs further investigations in the future.

## 3 Conclusion

Herein, we applied a sophisticated chiral molecule‐induced self‐assembly method to synthesize novel CHA‐coated Ti alloys, which possessed stereospecific L‐/D‐/Rac‐chiral hierarchical topography, improved hydrophilicity, and nanotopography rough surfaces. The different stereospecific chiral hierarchical structures exhibited highly enantioselective bone‐implants interactions, wherein L‐CHA strongly promoted osseointegration and vice versa for D‐CHA, thus resulting in a compromise outcome for Rac‐CHA. Of note, the present study highlights the superior enantiomer‐dependent osteoporotic osseointegration ability of L‐CHA, mainly by down‐regulating IL‐17 signaling pathway to manipulate desired immunomodulation coupled with enhanced neurovascularization. These results provide new insights into biological multifunctionality and mechanism underlying L‐chirality's roles for bone healing, thereby may inspiring the developments of new generation of chiral‐engineered biomaterials.

## 4 Experimental Section

### Substrate Treatment

Pristine titanium alloys (Ti‐6Al‐4 V, Φ 1.5 mm by 6 mm screws, Φ 20 mm by 1 mm disks) purchased from Biovet Yunao Co. (China) were soaked in deionized water for 30 min and then washed by acetone/ethanol (v/v = 5/5, Sinopharm) thrice under ultrasonic. Thereafter, pristine Ti samples were treated with dilute hydrochloric acid (1 m) solution for 2 h, washed again and dried for use.

### Preparation of CHA Coated Ti

The synthesis procedures of CHA were performed based on our previous researches.^[^ ^21^, ^22^ ^]^ Briefly, 1 mM (NH_4_)_2_HPO_4_ (Sinopharm) and 2.3 mm urea (Sinopharm) were dissolved in 23 mL ultrapure water to obtain P precursors, while 1 mm Ca(NO_3_)_2_·4H_2_O (Sinopharm) was dissolved in 5 mL ultrapure water to obtain Ca precursors. Moreover, 1 mm L‐, D‐, or Racemic tartaric acid (TTA, Tansoole) were respectively dissolved in 5 mL ultrapure water to obtain different chiral induction solutions, which were then mixed with Ca precursors. Subsequently, P precursors were added with vigorous stirring for 2 min. Then, Ti substrates were placed in the mixed solution with continuous stirring. After that, the solutions were transferred to an autoclave and subjected to adequate hydrothermal reaction under 180 °C for 24 h. Following reactions, the autoclave was cooled to collect CHA‐coated Ti, which were then washed thrice and dried overnight. The CHA‐coated Ti synthesized by L‐, D‐ and Rac‐TTA were named L‐CHA, D‐CHA, and Rac‐CHA respectively. In comparison, the achiral HA‐coated Ti synthesized without chiral induction solution was named HA for short. All synthetic materials were sterilized by ethylene oxide and stored for use.

### Materials Characterization

The surface morphological features of samples were detected using scanning electron microscopy (SEM‐EDS, JEOL, JSM‐7900F) at an accelerating voltage of 1.0 kV along with 4 mm working distance. In particular, the designated stereospecific chiral morphology (L‐, D‐ and Rac‐CHA) of samples were determined in quadruplicate. The phase structures were determined by X‐Ray Diffraction (XRD, Rigaku, D/MAX‐2200/PC) equipped with Cu Kα radiation (40 kV, λ = 0.15418 nm, 30 mA) under a scan rate of 1°/min within 10–80° diffraction angle. The specific chirality of CHA powders and coated Ti samples was detected using a diffuse reflectance ultraviolet‐visible (UV–vis) and circular dichroism (CD) spectropolarimeter (JASCO, J‐1500). Moreover, a typical pendant drop procedure was conducted to assess surface hydrophilicity using a contact angle analyzer (Solon, SL200B). Additionally, the surface roughness was observed by an atomic force microscopy (AFM, Bruker, Dimension Icon).

### OVX Rats Establishment and Assessment

The protocol of in vivo animal experiments was approved by the Institutional Animal Care and Use Committee of Shanghai Laboratory Animal Research Center (20231207‐001). Thirty‐five SD rats (female, 150–180 g) purchased from Shanghai Bikai Keyi Biotechnology Co., Ltd. were subjected to bilateral ovariectomy and fed 2 months to obtain ovariectomized (OVX) rats. To assess the osteoporosis condition of OVX rats, femurs of OVX/Sham rats (*n* = 4) were harvested and analyzed by micro‐CT (Skyscan 1275, Bruker). The percent bone volume (bone volume/tissue volume, BV/TV) and trabecular number (Tb.N) were calculated using the CTAn software. Then, samples were decalcified to prepare for serial transverse sections (1 mm proximal to the femoral growth plate) stained by immunohistofluorescence (NOS2, Arg‐1, CGRP, CD31, Abcam). All histological sections (*n* = 4) were detected by fluorescence microscope (Olympus CKX53) and the fluorescence intensity was analyzed using ImageJ software.

### In Vivo Bone Implantation Experiments

Thirty healthy OVX rats were randomly divided to five groups: (1) Ti, (2) HA, (3) L‐CHA, (4) D‐CHA, and (5) Rac‐CHA. A rat femoral condyle defect model was performed as shown in Figure 3A. Briefly, an incision ≈1.5 cm was made in the lateral knee joint to fully expose the bilateral femoral condyle, which was then properly drilled and inserted by sterilized Ti screws (1.5 × 6 mm, *n* = 4). Subsequently, the muscle, subcutaneous tissue, and skin were carefully sutured. At 1, 4, and 8 weeks post‐surgery, rats were sacrificed and the implants together with femurs were harvested for quantitative analysis.

### Evaluation of Implants Osseointegration

To assess the in vivo immunomodulation capacity, samples (*n* = 4) were harvested at 1 week post‐implantation and then dehydrated, embedded in methyl methacrylate for immunohistofluorescence staining (NOS2/Arg‐1, Abcam) at central areas of implants. For implants osseointegration evaluation, harvested samples (*n* = 4) were scanned by micro‐CT (Skyscan 1275, Bruker) and 3D reconstructed using the Dataviewer software. The parameters including percent bone volume (bone volume/tissue volume, BV/TV), bone mineral density (BMD), trabecular thickness (Tb.Th), and trabecular number (Tb.N) were quantified by the CTAn software. To analyze peri‐implant innervation, vascularization, and bone formation, samples were serially sectioned for H&E and immunohistofluorescence staining (CGRP, CD31, DAPI, Abcam) at central areas of implants. All sections (*n* = 4) were detected by fluorescence microscope (Olympus CKX53) and the percent bone‐implant contact (BIC) along with fluorescence intensity were quantified using ImageJ software.

### Cell Culture on Ti Samples

The macrophage cell line RAW 264.7 and human umbilical vein endothelial cells HUVECs were obtained from the National Collection of Authenticated Cell Cultures (Chinese Academy of Science). BMSCs were obtained from the femoral marrow cavity of OVX rats and cultured in α‐MEM (Gibco) containing 10% FBS (Gibco). After three cell passages, BMSCs were seeded (2 × 10^4^ cells mL^−1^) on each group of sterilized Ti disks (Φ 20 mm) for prolonged culture. For osteogenic differentiation assays, BMSCs were specifically supplemented with the commercial osteogenic induction medium (Cyagen). RAW cells were seeded (4 × 10^4^ cells mL^−1^) on each group of sterilized Ti disks (Φ 20 mm) and standardly cultured in DMEM (Gibco) containing 10% FBS (Gibco). HUVECs were seeded (2 × 10^4^ cells mL^−1^) on each group of sterilized Ti disks (Φ 20 mm) and standardly cultured in endothelial cell medium (ECM, ScienCell) containing 1% ECGS/H (Promocell) and 5% FBS (Gibco). The dorsal root ganglion (DRG) tissues were harvested from OVX rats and soaked in DMEM (Gibco) containing 10% FBS (Gibco). Subsequently, DRG tissues were fully tailored and digested with trypsin (Sigma) and collagenase A (Sigma). The isolated DRG sensory neurons were then centrifuged and resuspended in neurobasal A medium (Gibco) containing 2% B‐27 (Invitrogen), 1% L‐glutamine (LG, Gibco), and 1% penicillin/streptomycin (PS, Sigma). The obtained DRG cell suspension was seeded (3 × 10^4^ cells mL^−1^) on each group of sterilized Ti disks (Φ 20 mm) and cultured in neurobasal A/B‐27/LG/PS medium containing 20 ng mL^−1^ NGF (Sigma).

### DRG/HUVECs Cultured in RAW Conditioned Medium

RAW cells were seeded (4 × 10^4^ cells mL^−1^) on each group of sterilized Ti disks (Φ 20 mm) for 2 h and then induced to M1 polarization by 2.5 ng mL^−1^ IFNγ with 100 ng mL^−1^ LPS (Sigma) for 12 h. Subsequently, polarized RAW cells were continuously cultured for 5 days to collect supernatants, which were then centrifuged to obtain RAW‐conditioned medium (RAW‐CM). DRG neurons were seeded (3 × 10^4^ cells mL^−1^) on coverslips coated with poly‐d‐lysine hydrobromide (Sigma) and stimulated by RAW‐CM mixed (1:1 ratio) with neurobasal A/B‐27/LG/PS/NGF medium described above (Figure 5F). HUVECs were seeded in 24‐well (5 × 10^4^ cells mL^−1^) or 96‐well (2 × 10^4^ cells mL^−1^) plates and stimulated by RAW‐CM mixed (1:1 ratio) with complete ECM medium described above (Figure 6E).

### Cell Adhesion and Viability

Following 1 day culture on Ti disks, RAW cells were fixed by 2.5% glutaraldehyde for 1 h and then subjected to gradient dehydration using ethanol (50–70–90–100%) for 10 min each time. Subsequently, the adhesion of RAW cells was observed by SEM (JEOL, JSM‐7900F) and quantified via cell aspect ratios (*n* = 4) as previously performed.^[^ ^21^ ^]^ The adhesion of HUVECs/ DRG cultured on Ti disks was evaluated by actin cytoskeleton and nuclei staining. After cultured for 1 day, cell were washed with PBS twice and fixed by 4% paraformaldehyde. Thereafter, samples were permeabilized twice with PBS containing 0.1% Triton X‐100 (Sigma) for 5 min. Finally, cells were stained by phalloidin (Sigma). Cell spreading morphology was detected via confocal laser scanning microscope (Olympus FV1000) and then quantified via cell aspect ratios (*n* = 4). Additionally, following 3 days culture on Ti disks, the medium were removed and cells were washed by PBS thrice. Then, live/dead staining (Calcein AM/PI, 2 × 10^−3^ mmol L^−1^, Sigma) was conducted to observe the cell growth by fluorescence microscope (Olympus CKX53). Meanwhile, MTT (5 mg mL^−1^, Beyotime) assays were performed and the absorbances (OD value, 490 nm) were read using a microplate reader (Thermo Labsystems) to quantify cell viability (*n* = 4).

### Flow Cytometry Assays

RAW cells were seeded (4 × 10^4^ cells mL^−1^) on each group of sterilized Ti disks (*n* = 4) for 2 h and then induced by 2.5 ng mL^−1^ IFNγ with 100 ng mL^−1^ LPS (Sigma) for 12 h, followed by prolonged culture in standard medium for 3/7 days. Subsequently, polarized RAW cells were collected, centrifuged, and washed by PBS twice, followed by incubation with PE anti‐mouse CD11c antibody (M1 marker, Biolegend) and APC anti‐mouse CD206 antibody (M2 marker, Biolegend) for 30 min, respectively. The expression of M1/M2 markers was quantified by a flow cytometer (NovoCyte, Agilent).

### Immunofluorescence Staining

At designated culture time, cells were washed, fixed, permeabilized, and then blocked by normal goat serum (Beyotime) for 30 min. Subsequently, polarized RAW cells cultured on each group of Ti disks for 3/7 days were successively incubated with primary Arg‐1 antibody (Abcam) at 4 °C overnight and Alexa Fluor 488‐conjugated secondary antibody (Proteintech) for 60 min. RAW cells were washed, blocked by normal goat serum (Beyotime) again and further incubated with primary NOS2 antibody (Abcam) at 4 °C overnight and Alexa Fluor 594‐conjugated secondary antibody (Proteintech) for 60 min, respectively. Finally, RAW cells were incubated with DAPI (Proteintech) to stain nuclei. Likewise, after stimulated by RAW‐CM for 3 days in 24‐well plates, HUVECs were stained by CD31 antibody (Abcam) and DAPI (Proteintech). DRG neurons stimulated by RAW‐CM for 3 days were subjected to double marked immunofluorescence of NF200 (Proteintech)/CRGP (Santa Cruz)/DAPI (Proteintech) and NF200 (Proteintech)/Substance P (SP, Abcam)/DAPI (Proteintech) respectively. BMSCs were stained by osterix antibody (Abcam) and DAPI (Proteintech) following 3 days culture on each sample. Cells were then detected by fluorescence microscope (Olympus CKX53) and fluorescence intensity was determined (*n* = 4) via ImageJ software.

### RT‐PCR Assays

Total RNA of polarized RAW cells cultured on each group of Ti disks (*n* = 4) for 3/7 days was extracted using Trizol reagent (Invitrogen) and then reverse transcribed to cDNA by PrimeScript 1st Strand cDNA Synthesis Kit (Takara). RT‐PCR assay was conducted through the Bio‐Rad RT‐PCR system and gene expressions (TNFα, IL6, IL1β, Arg‐1, IL10) were normalized to GAPDH. Likewise, gene expressions (IL‐17A, IL‐17F, IL‐17B, IL‐17C) of RAW cells cultured on each sample for 5 days were analyzed by RT‐PCR assays. Primer sequences used are listed in Table S1 (Supporting Information).

### Transwell Assays

HUVECs were seeded (2 × 10^5^ cells mL^−1^) in upper chambers of 24‐well Transwell plates (Corning), and lower chambers were added with 600 µL mixed medium (RAW‐CM/complete ECM medium, 1:1) for 12 h incubation. Subsequently, HUVECs were washed, fixed, and stained by crystal violet (Beyotime) for 25 min. Thereafter, cells remained in upper chambers were wiped out and positive stained cells were counted to quantify cell migration (*n* = 4) under microscope (Olympus CKX53).

### Tube Formation Assays

96‐well plates were precooled on ice and coated with Matrigel (50 µL, BD Bioscience), followed by gelation under 37 °C for 60 min. Then, 100 µL HUVECs suspensions containing RAW‐CM and complete ECM medium (1:1) were seeded (1 × 10^5^ cells mL^−1^) on Matrigel and cultured for 4 h. Subsequently, HUVECs were observed and stained by calcein AM (Sigma) to calculate the branch points and tube length (*n* = 4) by fluorescence microscope (Olympus CKX53).

### Western Blot Assays

After stimulated by RAW‐CM for 3 days in 24‐well plates, HUVECs were washed by TBST (Beyotime) thrice and lysed by RIPA Lysis Buffer containing protease and phosphatase inhibitor (Beyotime) on ice, followed by 5 min centrifugation to extract proteins. The obtained proteins were transferred onto PVDF membranes (Millipore), blocked by 5% nonfat milk and incubated overnight with primary antibodies against VEGF (Abcam), eNOS (Abcam), FGF2 (Abcam), and GAPDH (Abcam) at 4 °C, followed by incubation with anti‐rabbit secondary antibody (Proteintech) for 1 h. Likewise, DRG neurons cultured in RAW‐CM together with/without supplement of KN‐93 (1 × 10^−6^ M, Sigma) or KG‐501 (10 × 10^−6^ M, Sigma) for 3 days were subjected to western blot assays using antibodies against pCaMKII (Santa Cruz), CaMKII (Santa Cruz), pCREB (Affinity), CREB (Affinity), CGRP (Santa Cruz), SP (Affinity) and GAPDH (Abcam). BMSCs cultured on each sample without/with supplement of MK2206 (5 × 10^−6^ M, Sigma) for 7/14 days were subjected to western blot assays using antibodies against ALP (Abcam), OCN (Affinity), RUNX2 (Abcam), pAKT (Abcam), AKT (Abcam) and GAPDH (Abcam). RAW cells cultured on each sample for 5 days were subjected to western blot assays using antibodies against IL‐17C (Affinity), TRAF6 (Affinity), pNF‐κB (Affinity), NF‐κB (Affinity), Mmp9 (Affinity) and GAPDH (Abcam). An enhanced chemiluminescence detection system (Beyotime) was applied to observe protein bands on PVDF membranes, and the gray value of each band was analyzed by the AlphaEaseFC software to determine relative protein expressions (*n* = 4).

### Ca^2+^ Fluorescence Probe Detection

Following 3 days culture on each group of Ti disks (*n* = 4), DRG neurons were washed by PBS thrice and stained by DAPI (Proteintech) for 10 min, followed by incubation with Hanks’ balanced solution (Beyotime) containing 4 µm Fluo‐4 AM (Sigma) and 1 µm Pluronic F‐127 (Sigma). Then, DRG neurons were washed by PBS again and further incubated in Hanks’ balanced solution (Beyotime) under 37 °C for 20 min. Intracellular Ca^2+^ in DRG neurons was detected by fluorescence microscope (Olympus CKX53) and then determined by ImageJ software. Likewise, Intracellular Ca^2+^ in DRG neurons stimulated by RAW‐CM for 3 days was also quantified (*n* = 4) by ImageJ software.

### Microfluidics Assays

A commercial microfluidic silicone device (Xona) consisting of triple compartments (1 central compartment and 2 outer compartments) and 500 µm microgroove barriers was applied to perform microfluidics assays. DRG neurons were seeded in central compartments at a density of 10^4^/cm^2^, and the outer compartments were then added with RAW‐CM of each group 4 h later. Following 3 days co‐culture in the microfluidic device, DRG neurons were subjected to immunofluorescence staining using TUBB3 antibody (Abcam) and DAPI (Proteintech). Finally, the DRG neurons axonal outgrowth was detected via fluorescence microscope (Olympus CKX53).

### ELISA Assays

After stimulated by RAW‐CM for 3 days, proteins in DRG neurons were extracted by RIPA Lysis Buffer containing protease and phosphatase inhibitor (Beyotime) as mentioned above. Then CGRP ELISA kit (Phoenix Pharmaceuticals) and SP ELISA kit (Abcam) were respectively used to measure CGRP and SP concentrations in supernatant samples (*n* = 4).

### Alizarin Red S Staining

BMSCs were cultured on each sample and incubated for 14 days using the osteogenic induction medium (Cyagen). Thereafter, the medium were removed to wash cells three times with PBS, and a commercial Alizarin Red S (ARS) staining kit (Beyotime) was applied to evaluate the production of mineralization nodules.

### RNA‐Seq Analysis

As shown in Figure 8A, total RNA of polarized RAW cells cultured on each group of Ti disks for 5 days was extracted via Trizol reagent (Invitrogen) and screened by NanoDrop (Thermo Fisher) and 2100 Agilent Bioanalyzer (Agilent) to obtain high‐quality RNA samples (*n* = 3). Then, RNA‐seq was conducted through DNBSEQ platform (Beijing Genomics Institute). Differentially expressed genes (DEGs) among groups were determined by a reliable threshold (adjusted *p*‐value < 0.05, fold change > 1.5), then analysis of enriched Gene Ontology (GO) and KEGG pathway was carried out via the Dr. Tom online platform (https://biosys.bgi.com). Furthermore, Gene set enrichment analysis (GSEA) was conducted to clarify the significant pathways using GSEA v4.3.2 application (https://www.gsea‐msigdb.org).

### Statistical Analysis

All data were presented as mean ± standard deviation. The statistical analysis was conducted using Student's *t*‐test and one‐way variance analysis (ANOVA) method with Graphpad Prism 8 software. Calculated *p* value < 0.05 was considered significantly different (^*^ *p* < 0.05, ^**^ *p* < 0.01).

## Conflict of Interest

The authors declare no conflict of interest.

## Supporting information



Supporting Information

## Data Availability

The data that support the findings of this study are available from the corresponding author upon reasonable request.
