# Supplementary material for: Chirality‐Induced Hydroxyapatite Manipulates Enantioselective Bone‐Implant Interactions Toward Ameliorative Osteoporotic Osseointegration
Source: Adv Sci (Weinh). 2024 Dec 31;12(8):2411602. doi: 10.1002/advs.202411602 (PMC11848601; doi:10.1002/advs.202411602)
Supplement: Supplementary file 1 — Supporting Information [file ADVS-12-2411602-s001.docx]

Supporting Information

**Chirality-induced hydroxyapatite manipulates enantioselective bone-implant interactions** **towards ameliorative osteoporotic osseointegration**

*Liang Yang, Jinzhou Du,* *Shengyang Jin, Shuyi Yang,* *Zhaowei Chen, Shiyang Yu, Cunyi Fan,* Chao Zhou,* and Hongjiang Ruan**

**Table S1.** Premier sequences used in RT-PCR


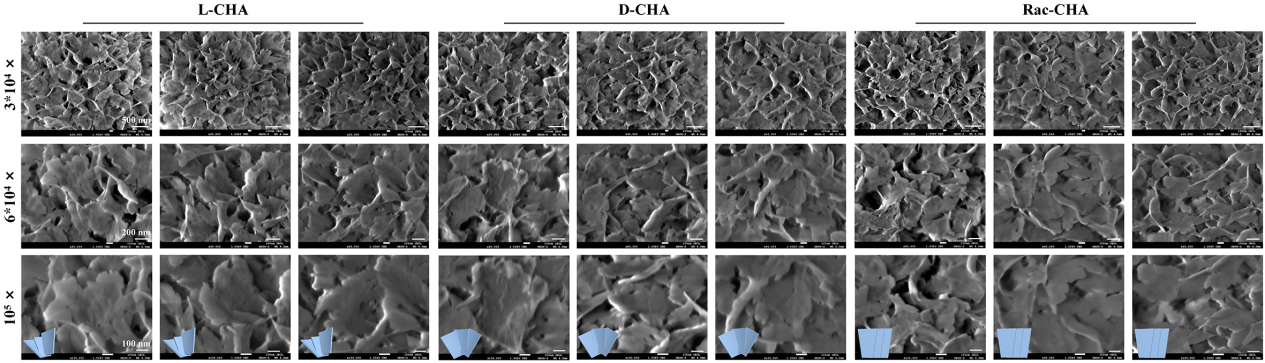


**Figure S1.** Representative chiral morphology (L-, D- and Rac-CHA) of samples observed by SEM.

| Gene | Forward Sequence | Reverse Sequence |
| --- | --- | --- |
| TNFα | CTGAACTTCGGGGTGATCGG | GGCTTGTCACTCGAATTTTGAGA |
| IL6 | ATAGTCCTTCCTACCCCAATTTCC | GATGAATTGGATGGTCTTGGTCC |
| IL1β | TGGAGAGTGTGGATCCCAAG | GGTGCTGATGTACCAGTTGG |
| Arg-1 | GGAATCTGCATGGGCAACCTGTGT | AGGGTCTACGTCTCGCAAGCCA |
| IL10 | GAGAAGCATGGCCCAGAAATC | GAGAAATCGATGACAGCGCC |
| IL-17A | TTTAACTCCCTTGGCGCAAAA | CTTTCCCTCCGCATTGACAC |
| IL-17F | TGCTACTGTTGATGTTGGGAC | AATGCCCTGGTTTTGGTTGAA |
| IL-17B | CACCCCCGGAACACCAAAG | CATACTCTTCCATTCGAGCGTAG |
| IL-17C | CTCCTGCTTCTAGGCTGGTTG | CCACCTGGCACTTCGAGTTAG |


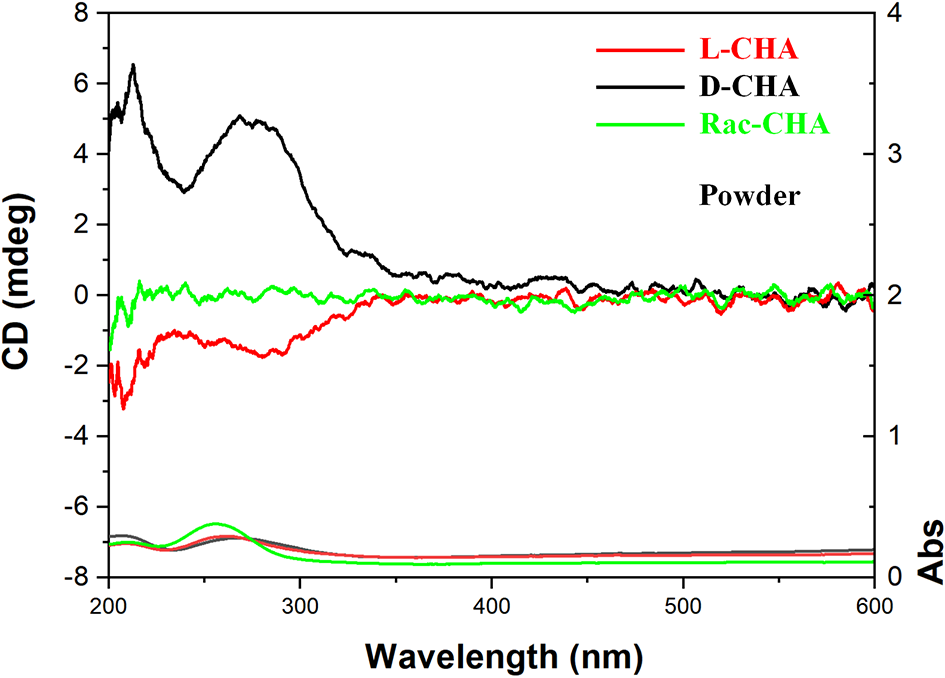


**Figure S2.** Chirality of CHA powders determined by UV-Vis and CD spectra.


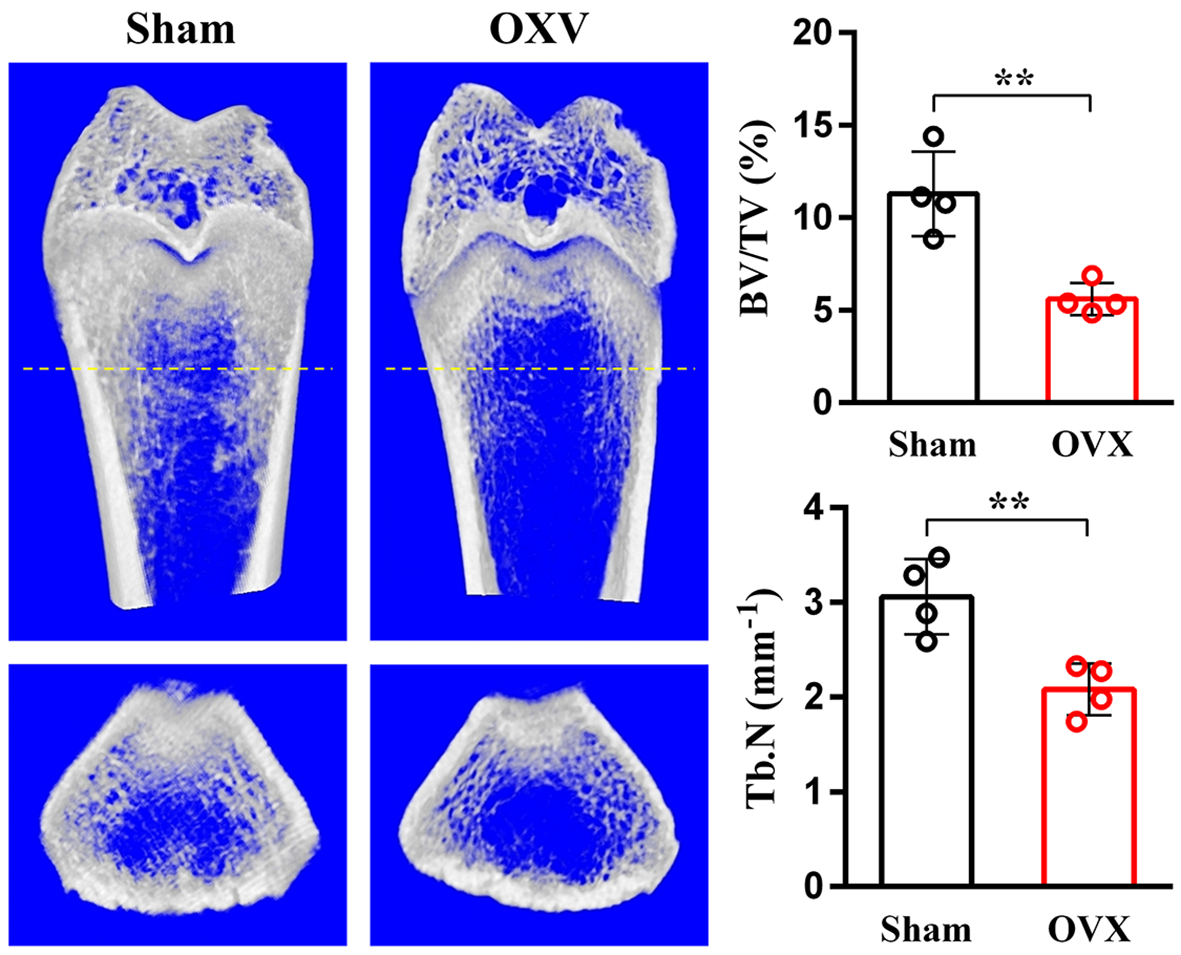


**Figure S3.** Osteoporotic condition evaluation following ovariectomy by micro-CT. Reconstructed coronal and transverse micro-CT images, and quantitative histormorphometry (BV/TV, Tb.N) of distal femurs in Sham and OVX rats. Data are presented as mean ± SEM. *n* = 4. ^**^P < 0.01.


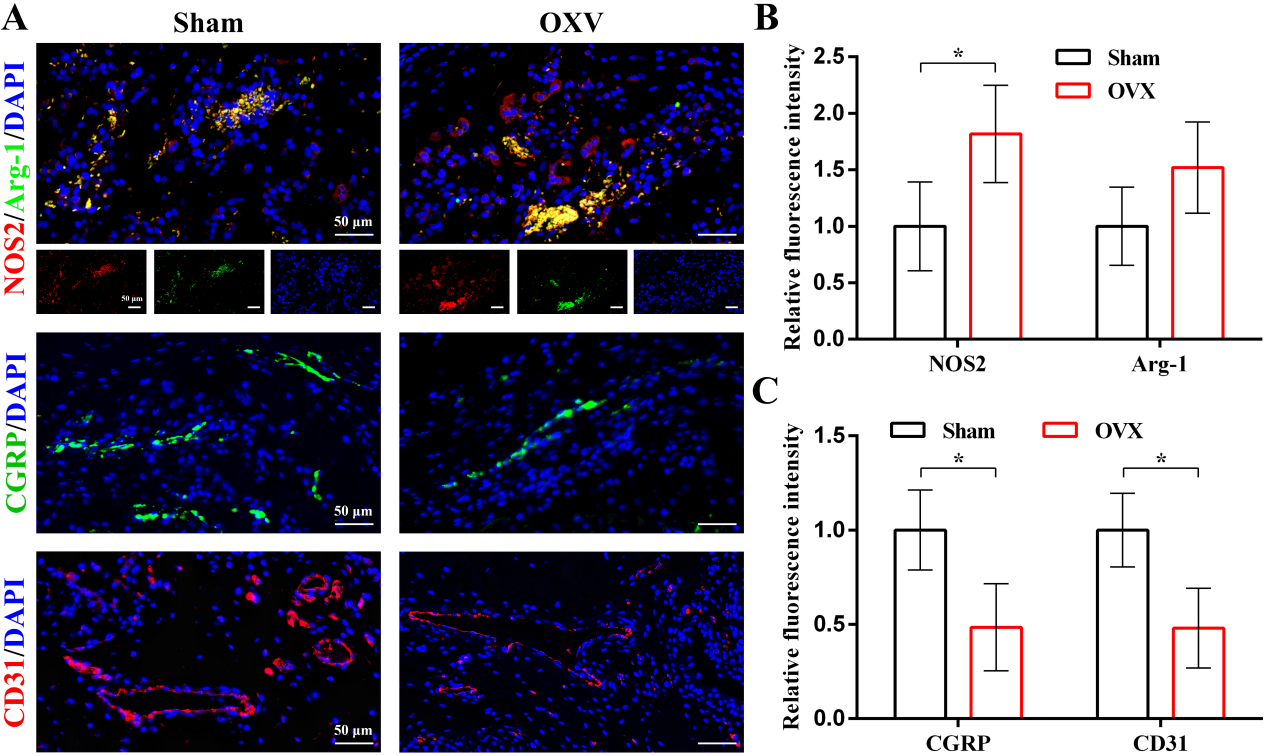


**Figure S4.** Osteoporotic condition evaluation following ovariectomy by histological analysis. (A) Representative immunofluorescent images of NOS2/Arg-1 (first and second row), CGRP (third row) and CD31 (fourth row) in Sham and OVX rats. (B and C) Quantitative fluorescence intensity of (B) NOS2, Arg-1 and (C) CGRP, CD31. Data are presented as mean ± SEM. *n* = 4. ^*^*P* < 0.05.


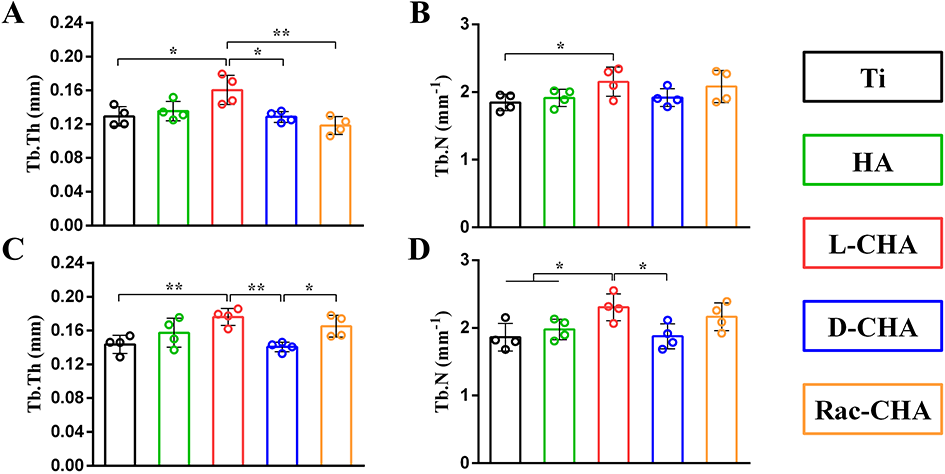


**Figure S5.** Quantitative analysis of micro-CT for bone-implant osseointegration. Quantification of micro-CT data in terms of (A and C) Tb.Th and (B and D) Tb.N at 4 and 8 weeks post-implantation respectively. Data are presented as mean ± SEM. *n* = 4. ^*^*P* < 0.05, ^**^*P* < 0.01.


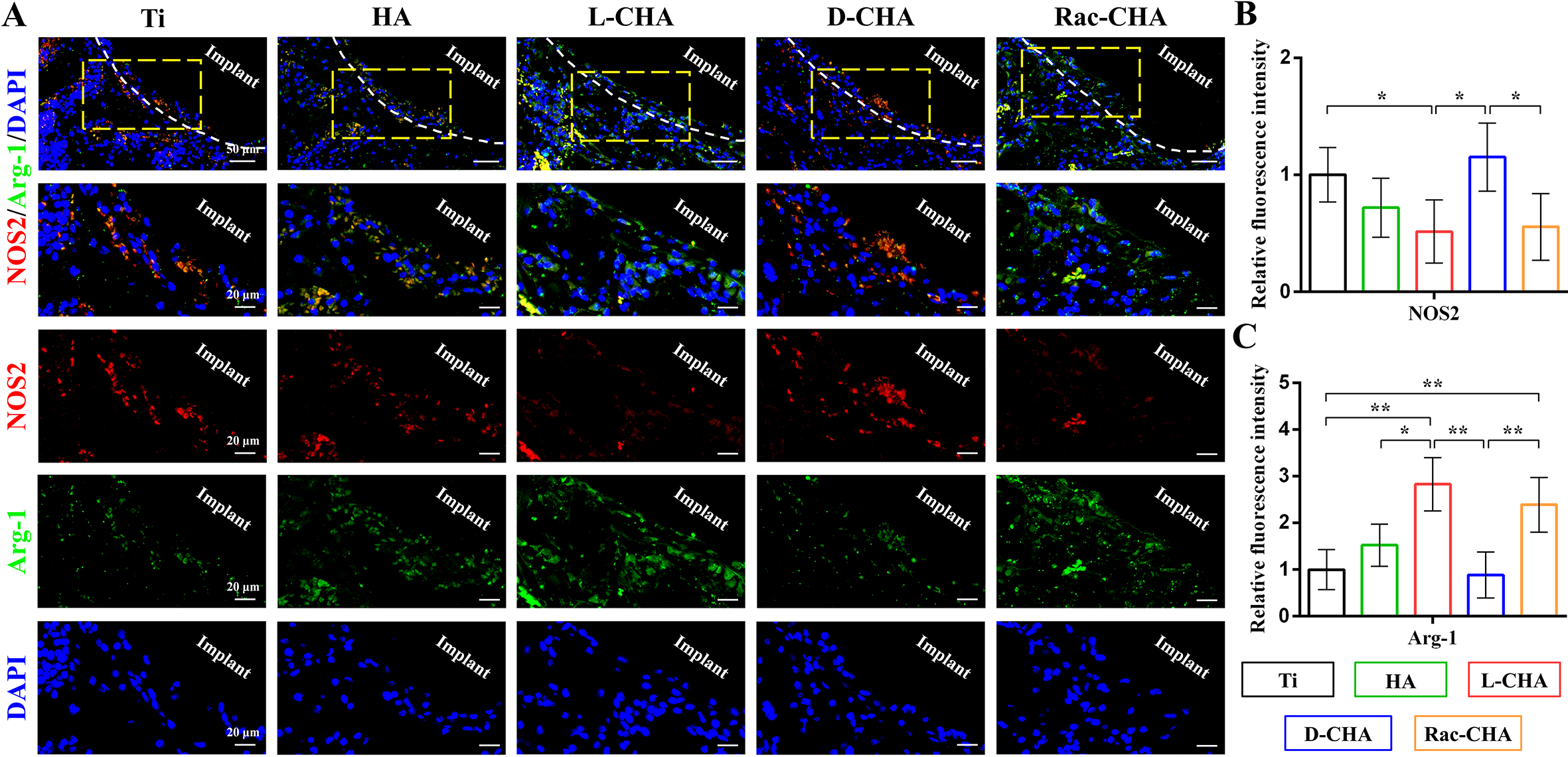


**Figure S6.** In vivo immunomodulation evaluation following 1 week implantation. (A) Median transverse sections of samples stained by immunohistofluorescence of NOS2/Arg-1. (B and C) Quantitative fluorescence intensity of (B) NOS2 and (C) Arg-1. The areas marked by yellow box are magnified for observation; White dashed lines indicate bone-implant interface. Data are presented as mean ± SEM. *n* = 4. ^*^*P* < 0.05, ^**^*P* < 0.01.


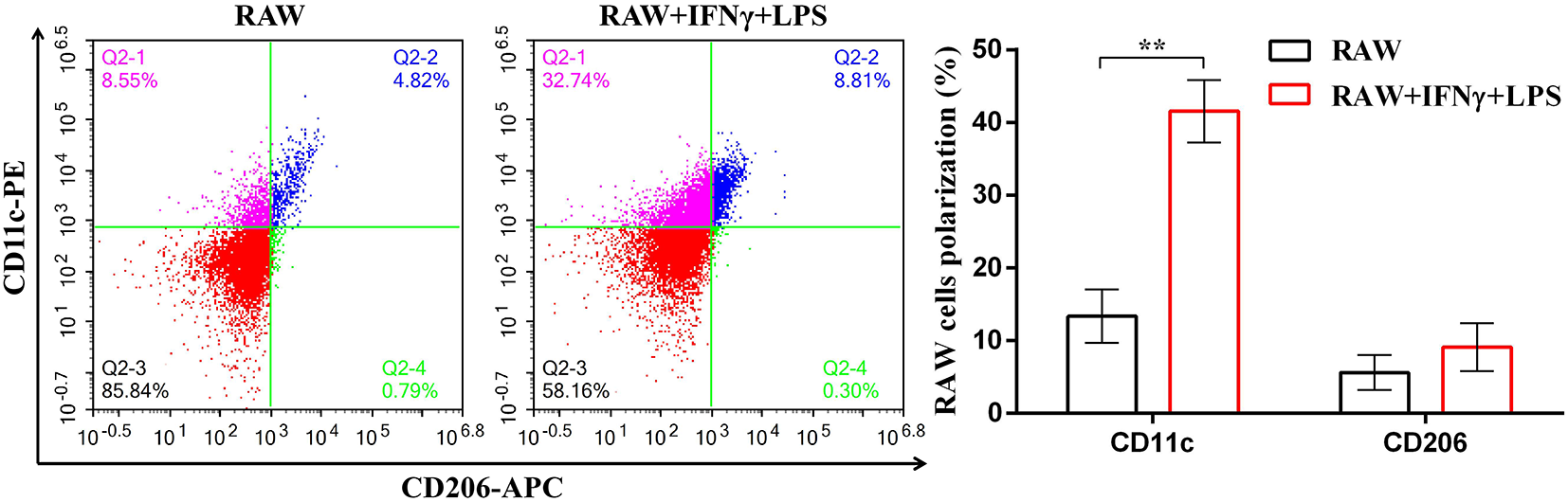


**Figure S7.** Flow cytometry evaluation of polarized RAW cells induced by IFNγ and LPS treatments. Data are presented as mean ± SEM. *n* = 4. ^**^*P* < 0.01.


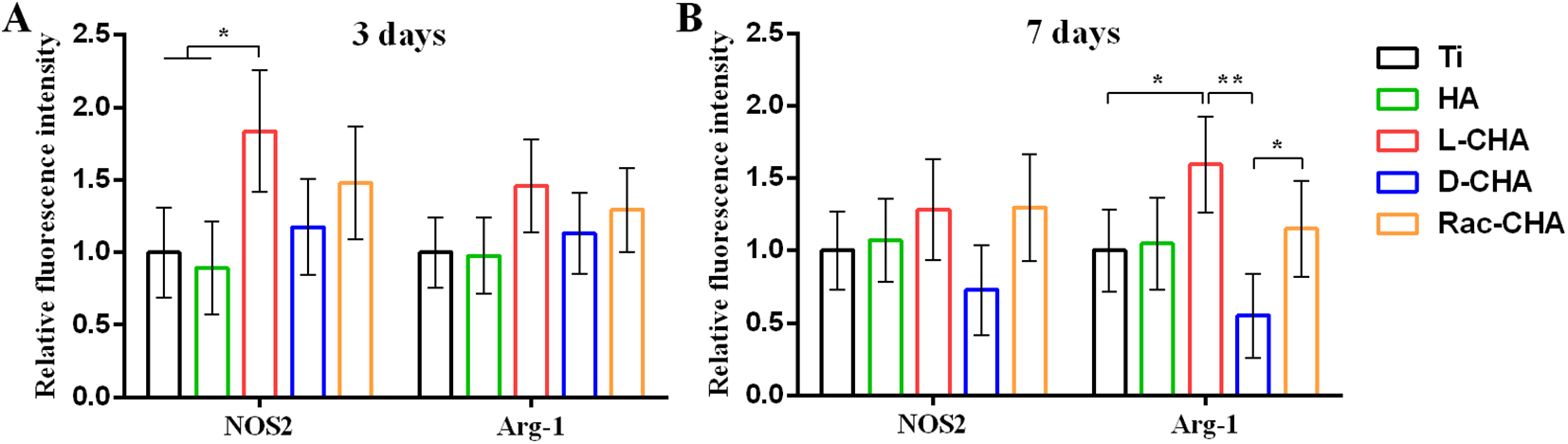


**Figure S8.** Quantitative fluorescence intensity of NOS2 and Arg-1 expressed by RAW cells cultured on samples for 3 and 7 days. Data are presented as mean ± SEM. *n* = 4. ^*^*P* < 0.05, ^**^*P* < 0.01.


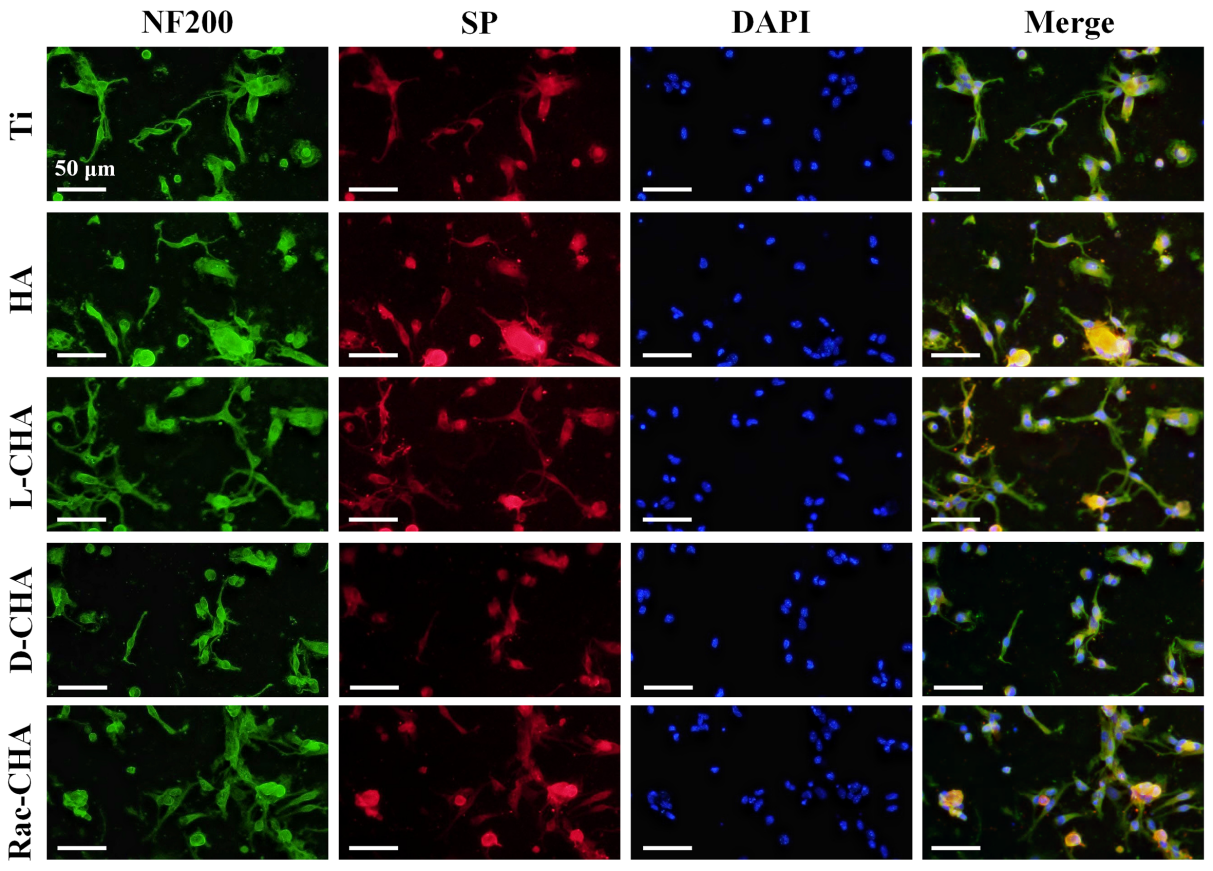


**Figure S9.** Immuno-inductive SP and NF200 expressions detected by immunofluorescence co-staining.


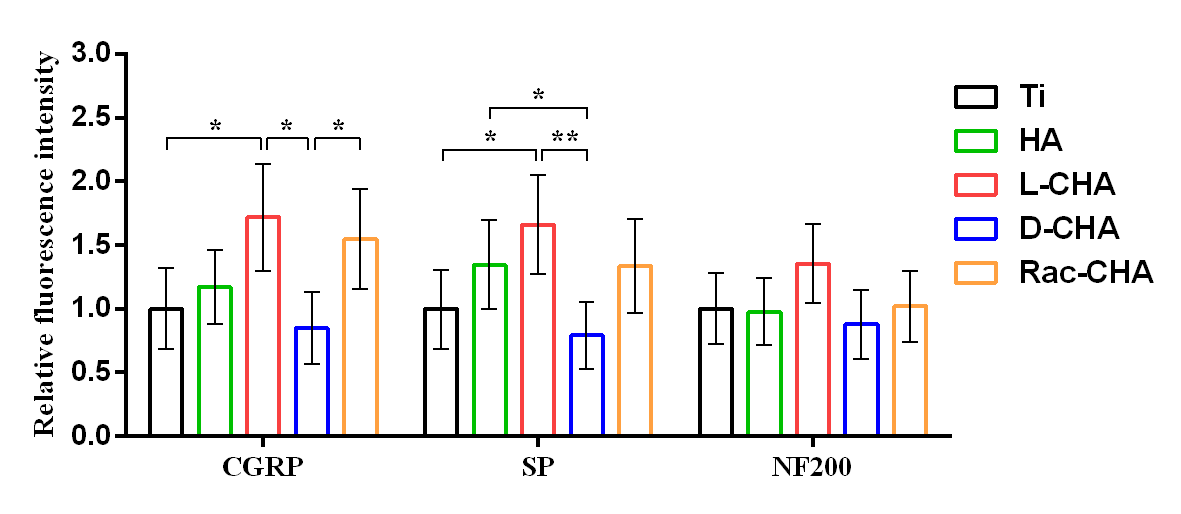


**Figure S10.** Fluorescence intensity quantitation of CGRP, SP and NF200 expressed by DRG neurons stimulated by RAW-CM for 3 days. Data are presented as mean ± SEM. *n* = 4. ^*^*P* < 0.05, ^**^*P* < 0.01.


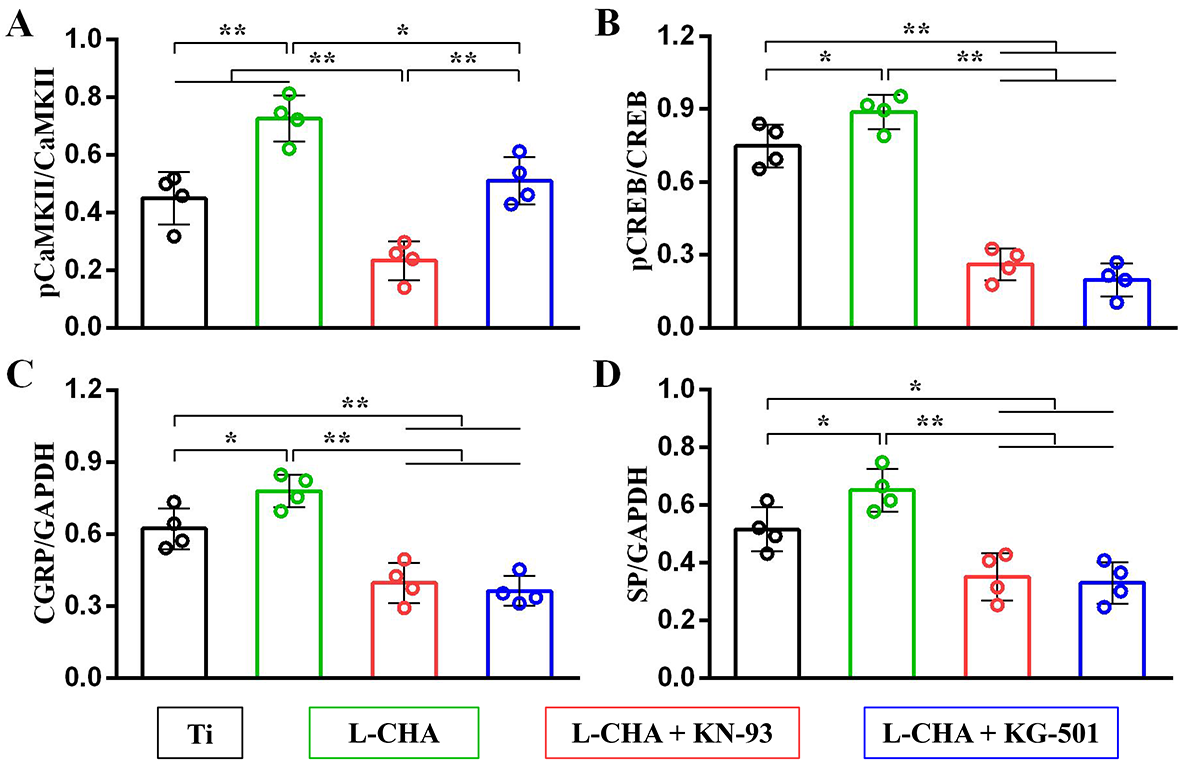


**Figure S11.** Western blot analysis of pCaMKII/CaMKII, pCREB/CREB, CGRP and SP expressions in DRG neurons cultured in RAW-CM supplemented with/without KN-93 or KG-501. Data are presented as mean ± SEM. *n* = 4. ^*^*P* < 0.05, ^**^*P* < 0.01.


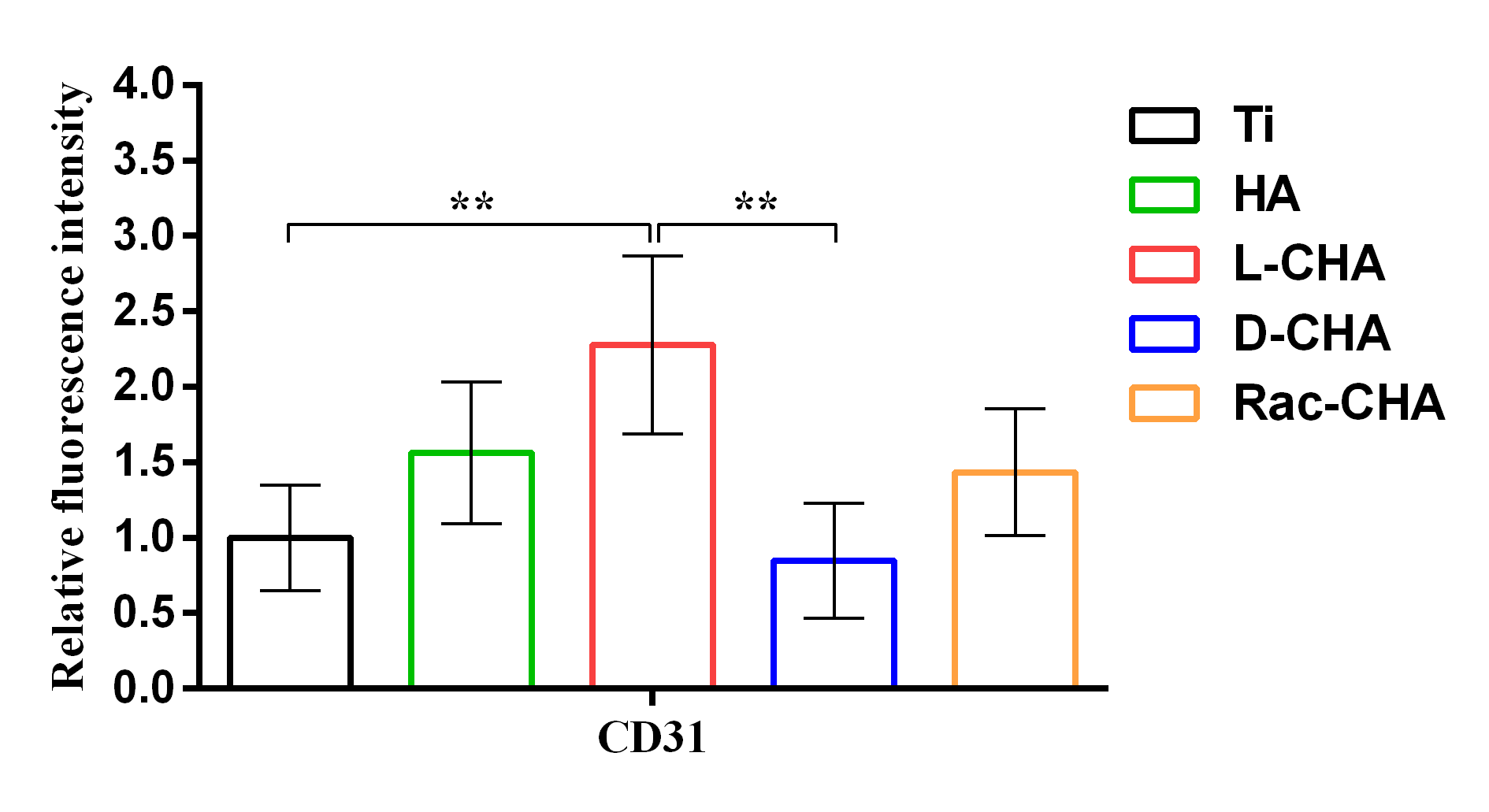


**Figure S12.** Quantitative fluorescence intensity of CD31 expressed by HUVECs stimulated by RAW-CM for 3 days. Data are presented as mean ± SEM. *n* = 4. ^*^*P* < 0.05.


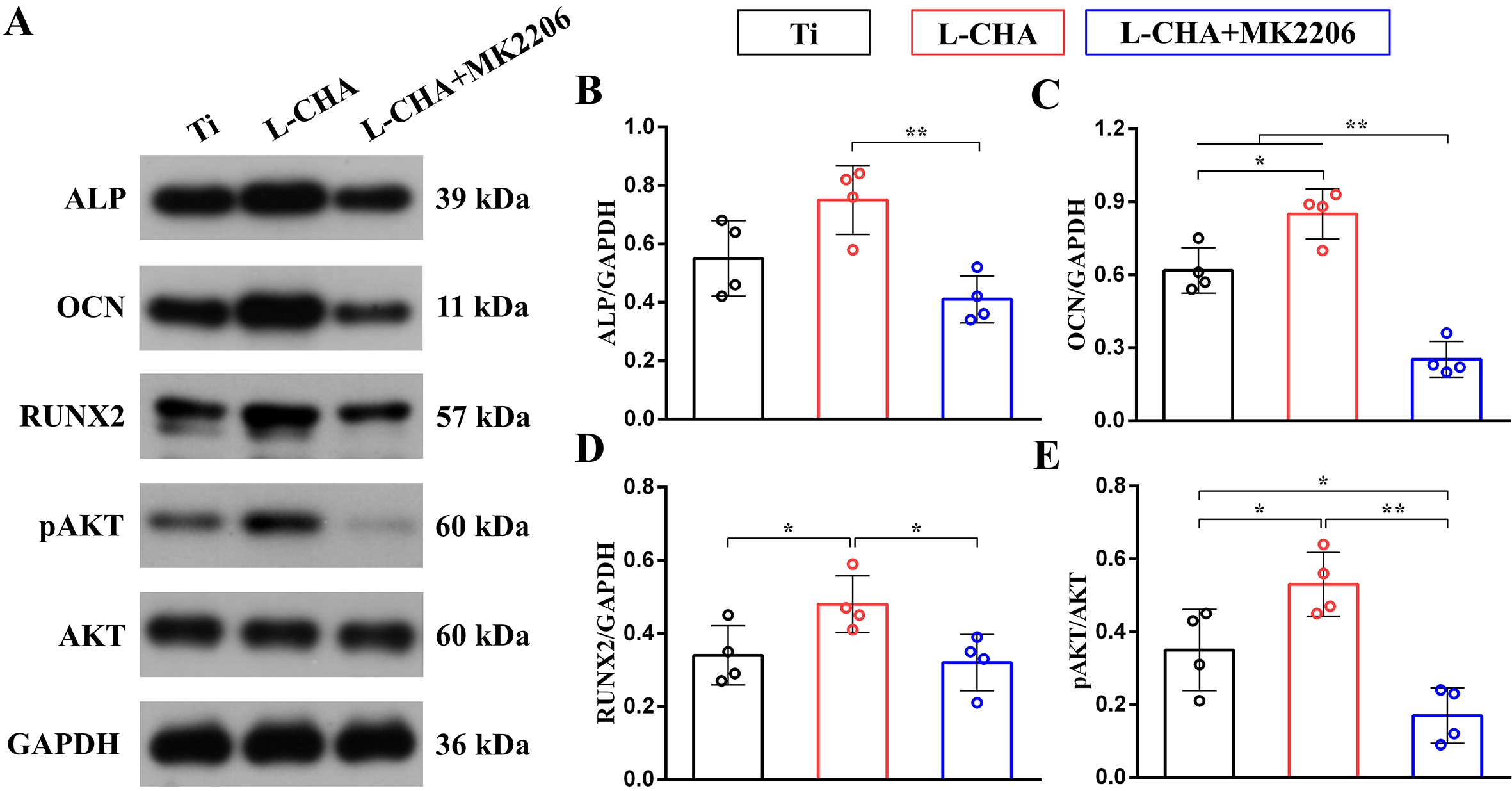


**Figure S13.** Western blot analysis of osteogenic markers (ALP, OCN, RUNX2, pAKT) expressions in L-CHA supplemented with/without MK2206 for 14 days culture. *n* = 4. ^*^*P* < 0.05, ^**^*P* < 0.01.


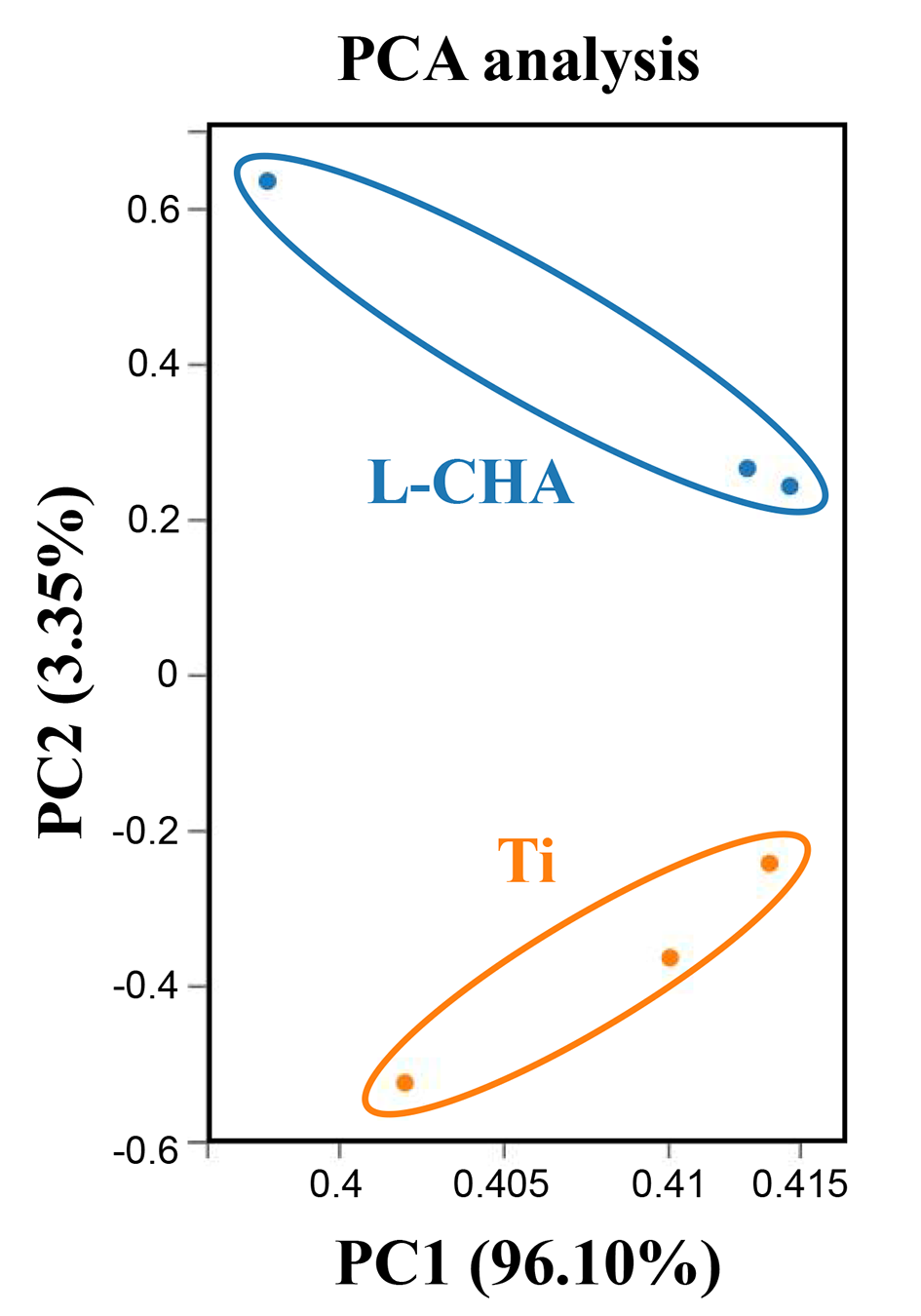


**Figure S14.** PCA analysis of the samples in L-CHA and pristine Ti for RNA-seq.


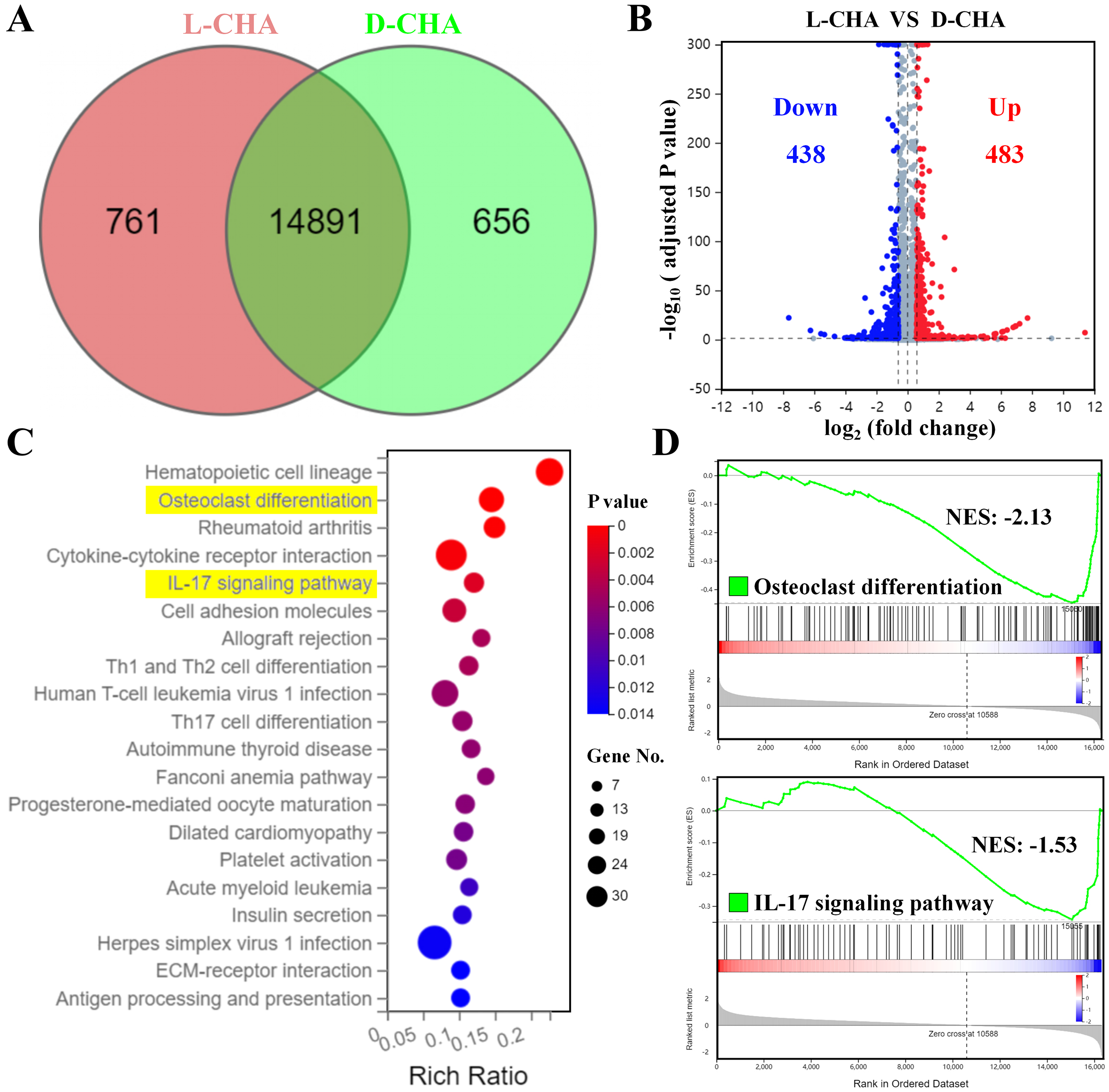


**Figure S15.** Transcriptomic RNA-Seq analysis between L-CHA and D-CHA groups. (A) Venn plot of the genes expression profile between L-CHA and D-CHA. (B) Volcano plot of DEGs in L-CHA versus D-CHA. (C) Enriched KEGG pathways based on DEGs of L-CHA versus D-CHA. (D) GSEA analysis for significant pathways (Osteoclast differentiation and IL-17 signaling pathway) down-regulated by L-CHA versus D-CHA. The yellow marks highlight interested KEGG pathways.


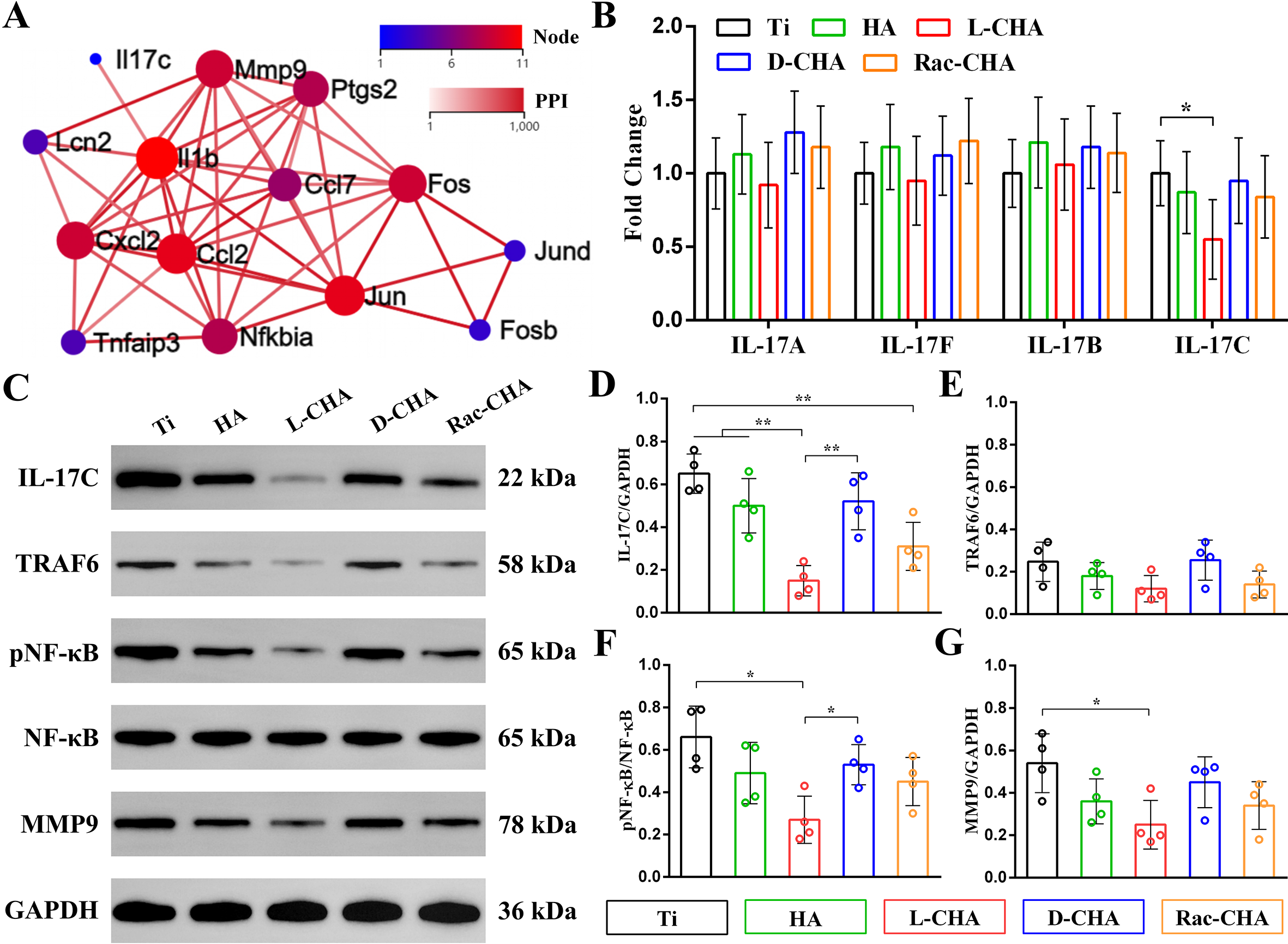


**Figure S16.** Signaling molecule analysis for the down-regulated IL-17 signaling pathway of RAW cells cultured on L-CHA. (A) PPI network and key genes involved in the down-regulated IL-17 signaling pathway of L-CHA versus pristine Ti. (B) IL17 family gene expressions determined by RT-PCR. (C) Western blot analysis of key signaling molecules involved in IL-17 signaling pathway. (D to G) Quantification of (D) IL-17C, (E) TRAF6, (F) pNF-κB and (G) MMP9 expressions based on western blot results. *n* = 4. ^*^*P* < 0.05, ^**^*P* < 0.01.


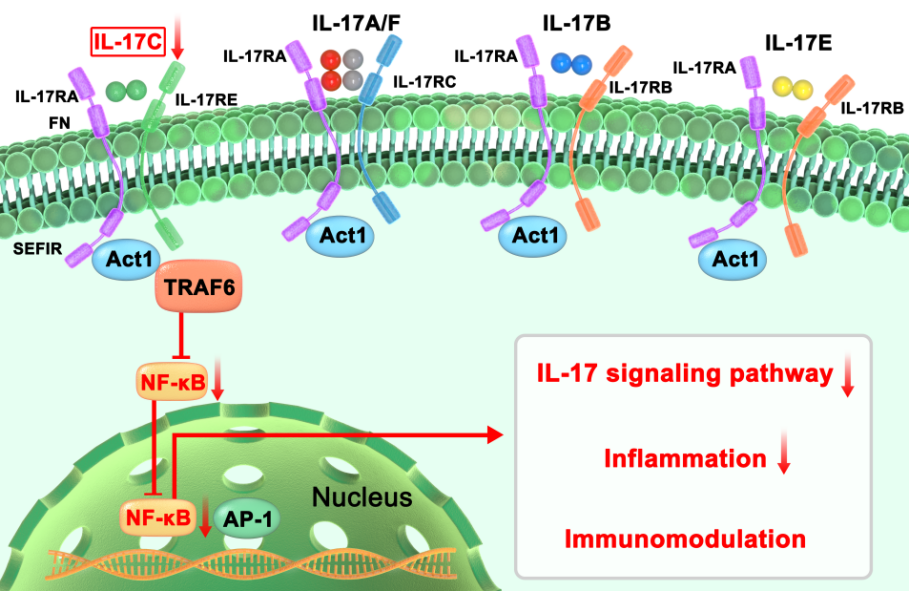


**Figure S17.** The underlying signaling transduction process of IL-17 signaling pathway down-regulated by L-CHA.
